# TGIF2-mediated HMGB3 overexpression promotes esophageal squamous cell carcinoma proliferation and metastasis through TLR3/TGF-β signaling

**DOI:** 10.1016/j.gendis.2025.101987

**Published:** 2025-12-15

**Authors:** Liaoran Niu, Wanli Yang, Wei Zhou, Lili Duan, Qi Wang, Xiaoqian Wang, Yiding Li, Chengchao Xu, Yujie Zhang, Jinqiang Liu, Jian Zhang, Daiming Fan, Jianyong Zheng, Liu Hong

**Affiliations:** a Department of Digestive Surgery, Xijing Hospital, Fourth Military Medical University, Xi’an, Shaanxi 710032, China; b State Key Laboratory of Holistic Integrative Management of Gastrointestinal Cancers and National Clinical Research Center for Digestive Diseases, Xijing Hospital of Digestive Diseases, Fourth Military Medical University, Xi’an, Shaanxi 710032, China; c Department of Dermatology, Xijing Hospital, Fourth Military Medical University, Xi’an, Shaanxi 710032, China; d 94719 Military Hospital, Ji’an, Jiangxi 343000, China; e Department of Histology and Embryology, School of Basic Medicine, Xi’an Medical University, Xi’an, Shaanxi 710032, China; f The State Key Laboratory of Cancer Biology, Department of Biochemistry and Molecular Biology, Fourth Military Medical University, Xi’an, Shaanxi 710032, China

**Keywords:** ESCC, HMGB3, Metastasis, Proliferation, TGIF2

## Abstract

Proliferation and metastasis are the core malignant characteristics in esophageal squamous cell carcinoma (ESCC) that contribute to poor prognosis. However, the mechanisms underlying cell proliferation and metastasis remain elusive. We explored the function of high-mobility group box (HMGB3) in promoting ESCC progression. HMGB3 expression in ESCC tissues and cell lines was quantified using quantitative PCR, Western blotting, and immunohistochemistry. The proliferative and migratory characteristics of ESCC cells were assessed using *in vitro* and *in vivo* assays, respectively. An RNA sequencing analysis was conducted to identify the downstream signaling pathways of HMGB3. Co-immunoprecipitation was performed to identify HMGB3-interacting proteins. HMGB3 transcriptional regulation was investigated using luciferase reporter and chromatin immunoprecipitation assays. Elevated levels of HMGB3 were observed in both patient-derived ESCC tissues and ESCC cell lines and were correlated with poor patient prognosis. HMGB3 up-regulation promoted ESCC proliferation and metastasis, whereas HMGB3 down-regulation inhibited these processes. Mechanistically, homeodomain protein transforming growth factor beta (TGF-β)-induced factor homeobox 2 (TGIF2) transcriptionally up-regulates HMGB3. HMGB3 subsequently activates TGF-β signaling through its regulation of and interaction with toll-like receptor 3 (TLR3), ultimately promoting ESCC proliferation and metastasis. Clinically, HMGB3 expression was positively correlated with TGIF2 and TGF-β, and patients with ESCC who positively co-expressed TGIF2/HMGB3, HMGB3/TGF-β, or TGIF2/TGF-β exhibited poor prognosis. The functional role of HMGB3 in ESCC proliferation and metastasis was illustrated in our research. Targeting the TGIF2/HMGB3/TLR3/TGF-β axis has the potential to serve as a promising therapeutic approach.

## Introduction

ESCC is the seventh most prevalent cancer worldwide. In addition, it ranks sixth in mortality among all cancer types.^1^^,^^2^ Despite advances in early diagnosis and effective treatment, the prognosis for long-term survival among patients with ESCC remains poor, owing to uncontrolled proliferation and distant metastases.^3^ The prognosis of ESCC remains poor, primarily because most patients are diagnosed at an advanced stage. With a 5-year survival rate of just 15% in such cases, achieving long-term survival poses a significant challenge.^1^^,^^4^ Numerous studies have investigated the mechanisms underlying ESCC progression; however, they have been largely unsatisfactory.^5^ Therefore, more comprehensive research is necessary to uncover the mechanisms underlying ESCC development and progression and establish new therapeutic approaches.

HMGB3 is a member of the HMGB protein family, a class of non-histone nuclear proteins involved in fundamental processes like cell differentiation, proliferation, and death.^6^^,^^7^ Unlike the well-documented oncogenic roles of HMGB1 and HMGB2.^8^^,^^9^ HMGB3 has been relatively overlooked in cancer research since its initial discovery in B-cell differentiation.^10^ This disparity in research focus highlights a significant knowledge gap.^9^^,^^10^ Nevertheless, recent studies have rapidly expanded our understanding, revealing that HMGB3 promotes cancer progression through key mechanisms including angiogenesis,^11^ drug resistance,^12^^,^^13^ radioresistance,^14^ and immune escape.^15^ High HMGB3 levels have been observed in digestive system tumors, including gastric cancer,^16^ hepatocellular carcinoma,^17^ and ESCC.^18^ Our previous study has revealed that HMGB3 is up-regulated in ESCC tissues, indicating its potentially significant role in its progression.^19^ HMGB3 promotes the malignant progression of ovarian cancer cells by activating the mTOR signaling pathway.^12^ Additionally, HMGB3 can selectively bind to matrix metallopeptidase 7 (MMP7) and facilitate distant metastasis in colorectal cancer.^20^ However, the specific roles and mechanisms of HMGB3 in ESCC proliferation and metastasis have yet to be elucidated, highlighting the urgent need for an in-depth investigation. Drawing upon these findings and integrating insights from previously published studies, we investigated the upstream and downstream regulatory mechanisms of HMGB3 in ESCC development.

To elucidate the upstream regulatory mechanism of HMGB3, we employed the JASPAR database to predict potential transcription factors binding to the HMGB3 promoter. This analysis identified TGF-β-induced factor homeobox 2 (TGIF2) as a potential candidate. TGIF2 belongs to the three-amino acid loop extension (TALE) superfamily.^21^^,^^22^ Up-regulated in different tumors, including lung adenocarcinoma,^23^ melanoma,^24^ and colorectal cancer,^25^ TGIF2 plays crucial roles in modulating tumor proliferation, invasion, and metastasis.^26^^,^^27^ The most extensively studied TGIF2 function involves recruiting histone deacetylases to DNA-bound Smad transcription complexes, thus regulating transforming growth factor beta (TGF-β) signaling.^28^ Additionally, TGIF2 acts as a transcription factor, regulating key genes such as fucosyltransferase 8 (FUT8)^24^ and octamer-binding transcription factor 4 (OCT4).^23^ However, its exact function in ESCC has remained unknown. We confirmed TGIF2 as a transcriptional regulator of HMGB3, partially unveiling the potential mechanism by which TGIF2/HMGB3 regulates the TGF-β signaling pathway in ESCC.

To investigate HMGB3’s downstream mechanisms, we performed RNA sequencing and selected toll-like receptor 3 (TLR3) as a candidate target based on differentially expressed genes and Kyoto Encyclopedia of Genes and Genomes (KEGG) pathway analysis. TLR3 is a member of the TLR family, which has previously been associated with HMGB1-mediated inflammation and carcinogenesis.^29^^,^^30^ It has been shown to positively regulate TGF-β signaling in various cancers, including neuroblastoma^31^ and breast cancer.^32^ Although TLR3 is known to be up-regulated in ESCC,^33^ its precise mechanistic role remains unclear. In this study, we reveal that HMGB3 may activate TGF-β signaling through interaction with TLR3 in ESCC, providing new insights into how HMGB3 modulates the TGF-β pathway.

Although previous studies have linked HMGB3 to ESCC,^18^^,^^19^ our study is the first to systematically investigate its specific functions and underlying mechanisms in ESCC tumorigenesis. Figure S1 depicts a flowchart of the study. Our study revealed a significant association between HMGB3 overexpression and the proliferation and metastasis of ESCC, and that its up-regulation serves as an adverse prognostic indicator for patients with ESCC. TGIF2, an upstream transcription factor of HMGB3, promotes ESCC progression by activating HMGB3 transcription via binding to its promoter. TGIF2-induced activation of the TGF-β signaling pathway is partially dependent on HMGB3. Additionally, TLR3 is a co-interacting molecule of HMGB3, and their combination can promote TGF-β signaling. Overall, targeting the TGIF2/HMGB3/TLR3/TGF-β axis could be a potential therapeutic strategy for ESCC.

## Materials and methods

### Cell culture and tissue collection

Het-1A, a human immortal esophageal squamous epithelial cell line, was provided by Zhengzhou University, China. Human ESCC cell lines EC109, ECA109, KYSE-150, and EC9706 were maintained in the laboratory (Xian, China). The primary ESCC cell was purchased from Meisen (Zhejiang, China). Cells were grown in the RPMI-1640 medium (Gibco; Massachusetts, USA) containing 10% fetal bovine serum (Oricell; Shenzhen, China) and antibiotics, including streptomycin (100 μg/mL) and penicillin (100 U/mL). Twenty pairs of ESCC and their matched paratumor tissues were procured from patients with ESCC who underwent radical resection at the Xijing Hospital of Digestive Diseases, Fourth Military Medical University. The samples were used for RNA expression detection using quantitative reverse transcriptase-polymerase chain reaction (qRT-PCR). Additionally, twenty paired paraffin-embedded pathological specimens of ESCC and matched adjacent tissues were selected from the Department of Pathology for the evaluation of TLR3 protein expression via immunohistochemistry (IHC). The inclusion and exclusion criteria were described in the supplementary materials and methods, and the clinical characteristics are summarized in Table S1. All human tissue collections were approved by the Institutional Review Board of Xijing Hospital, Fourth Military Medical University (approval No. KY20212195-F-1).

### Total RNA extraction and qRT-PCR

We used the PrimeScript RT Reagent Kit (TaKaRa; Tokyo, Japan) to isolate RNA from tissues and cells using the TRIzol reagent (Invitrogen; Waltham, Massachusetts, USA), and cDNA was synthesized. The expression of HMGB3, TGIF2, and TLR3 was assessed by qRT-PCR using the SYBR Premix Ex Taq II Kit (TaKaRa). The mRNA expression was normalized to GAPDH, serving as an internal standard, and calculated using the 2^–ΔΔCt^ method. The primer sequences are listed in Table S2.

### Western blotting (WB)

Radioimmunoprecipitation assay (RIPA) buffer (Beyotime Biotechnology; Shanghai, China) with phosphatase inhibitors (Millipore; California, USA) and protease inhibitors (Millipore, California, USA) was used to lyse the cells. Following centrifugation at 12,500 *g* for 15 min, supernatants were obtained. The total protein concentration was quantified using a BCA Protein Assay Kit (Pierce, Illinois, USA). Equal amounts of protein were separated using 10% sodium dodecyl sulfate-polyacrylamide gel electrophoresis (SDS-PAGE). Next, proteins were transferred to nitrocellulose membranes (Millipore; Temecula, California, USA). Afterward, before incubation with primary antibodies overnight at 4 °C, membranes were blocked with 5% non-fat milk at 37 °C for 1 h. Subsequently, membranes were treated with horseradish peroxidase-conjugated secondary antibodies against rabbit or mouse IgG (Abcam, Massachusetts, USA, 1:5000) at 37 °C for 1 h. The following primary antibodies were used to assess the expression of proteins: anti-β-actin (#3700; Cell Signaling Technology, Massachusetts, USA, 1:5000), anti-TGIF2 (#ab190152; Abcam, 1:1000), anti-p-TGIF2 and TGIF2 (#sc-390870; Santa Cruz, CA, USA), anti-HMGB3 (#ab75782; Abcam, 1:1000), anti-TLR3 (#ab62566; Abcam, 1:1000), anti-TGF-β (#ab215715; Abcam**,** 1:1000), anti-SMAD2/3 (#8685; Cell Signaling Technology, 1:1000), anti-SMAD2 (#5339; Cell Signaling Technology, 1:1000), anti-p-SMAD2 (#3108; Cell Signaling Technology, 1:1000), anti-SMAD3 (#9523; Cell Signaling Technology, 1:1000), anti-p-SMAD3 (#9520; Cell Signaling Technology, 1:1000), anti-extracellular signal-regulated kinase 1/2 (ERK1/2) (#4695; Cell Signaling Technology, 1:1000), anti-p-ERK1/2 (#4370; Cell Signaling Technology, 1:1000), anti-nuclear factor kappa-light-chain-enhancer of activated B cells (NF-kB) P65 (#8242S; Cell Signaling Technology, 1:1000), and anti-His-tag (#66005-1-Ig; protein-tech, 1:1000). Protein bands were quantified using ImageJ densitometry software, with β-actin serving as the loading control for normalization of target protein expression levels.

### Cell transfection and infection

HMGB3, TGF-β, P65 siRNA, and scrambled siRNA were designed and provided by RIBOBIO (Guangzhou, China). The siRNAs were transfected into cells using riboFECT™ CP (RIBOBIO, Guangzhou, China). The cells were subjected to related experiments after 48 h of culture. The siRNA sequences are listed in Table S3. A knockdown efficiency of 70% was established as the cutoff, and only siRNAs meeting this threshold were selected for use in further experiments.

GeneChem Co., Ltd. (Shanghai, China) designed and supplied the negative control and lentiviral vectors for the elevation or down-regulation of target genes. The specific shRNA sequences are listed in Table S3. ESCC cells were transfected with lentiviral vectors at a multiplicity of infection (MOI) of 40. Subsequently, indicated cells were subjected to selection with 1–4 μg/mL puromycin for 14 days.

### Cell counting kit-8 (CCK-8) assay

The cells were plated in 96-well plates at a density of 1000 cells in 100 μL per well, with cell count and viability (>90%) verified via trypan blue exclusion using the LUNA-II™ automated cell counter (Logos Biosystems) before plating. Every 24 h for 5 days, the original medium was substituted with a blend of 180 μL of fresh medium excluding fetal bovine serum and 20 μL of CCK-8 solution (Transgene; Beijing, China). Subsequently, the mixture was incubated at 37 °C for 3 h. The absorbance at 450 nm was assessed using a microplate reader (Bio-Rad; Hercules, California, USA).

### Colony formation

The ESCC cell suspension was plated in a 6-well plate at a density of 800 cells/well. The cells were incubated in a 5% CO_2_ environment at 37 °C for 2 weeks. Afterward, the cells were fixed with 10 % formalin for 20 min and stained with 0.1% crystal violet for 20 min. Colony numbers were quantified using ImageJ.

### Organoid culture and detection of organoid viability

Tumor tissues were collected, washed with advanced DMEM/F12 containing 1 × P/S, and minced into small fragments in digestion buffer (advanced DMEM/F12, 1 × P/S, 1 mg/mL Primocin, 0.6 mg/mL Collagenase, 20 mg/mL Hyaluronidase, and 10 mM Y-27632). After 1 h of digestion at 37 °C, the tissue suspension was filtered through an 80 μm cell strainer to remove debris. The cell clusters were then centrifuged at 350 *g* for 5 min, washed two times with DMEM/F12 culture medium, and resuspended in Matrigel (Corning, New York, USA) diluted 3:1 with phosphate-buffered saline (PBS). The suspension was plated in 24-well plates to form organoids, which were cultured in human esophageal cancer organoid medium (MA-0807T014L, Mogengel, Xiamen, China).

For passage, organoids were treated with trypsin for 3–5 min at 37 °C, washed, and re-embedded in Matrigel diluted 3:1 with PBS. Organoids were cryopreserved in cryotubes containing culture medium with 10% DMSO.

Organoids were dissociated into single cells using trypsin. Subsequently, 2000 cells/well were plated in 96-well plates and transfected with si-Control, si-HMGB3, or si-TGIF2. After 24 h, the medium was replaced with fresh culture medium, followed by 5 days of culture before viability assessment. Organoid viability was quantified using the CellTiter-Glo® 3D Assay (CTG, Promega, WI, USA) according to the manufacturer’s protocol. Briefly, the CTG reagent was diluted 1:10 in culture medium and added to each well. After 10 min of orbital shaking (500 rpm), plates were incubated at room temperature for 30 min to stabilize luminescent signals. Luminescence was measured using a microplate reader. Viability was calculated as a percentage relative to si-Control-transfected organoids.

### Assessment of *in vitro* cell migration and invasion

Cell migration and invasion abilities were assessed *in vitro* using 24-well Transwell inserts with 8 μm pores (Corning, NY, USA). Before the assay, cells were starved. For the invasion assay, the upper chamber was coated with 200 μg/mL Matrigel (Corning, NY, USA), and 8 × 10^4^ cells were seeded in serum-free medium. For the migration assay, 4 × 10^4^ cells in 200 μL of serum-free medium were added to the uncoated upper chamber. The lower chamber was filled with medium containing 20 % fetal bovine serum as a chemoattractant. After an appropriate incubation period, the number of cells that migrated or invaded through the membrane was counted.

### Cell cycle assay

ESCC cells with stable lentiviral infection, which had reached the logarithmic growth phase, were harvested by trypsinization and collected in a 1.5 mL centrifugation tube. After fixation in 75% ethanol for 1 h at 4 °C, the cells were washed with PBS and subsequently stained with 400 μL of propidium iodide (Servicebio; Wuhan, China) and 100 μL of RNase (BD Pharmingen; Guangzhou, China) in the dark at 37 °C for 30 min. The cell cycle was analyzed using a flow cytometer (CytoFLEX; Beckman Coulter, California, USA).

### Bromodeoxyuridine (BrdU) incorporation assay

BrdU (Sigma, Missouri, USA) was used at a final concentration of 10 μM. The cells were fixed in 4% paraformaldehyde and permeabilized for BrdU detection using an anti-BrdU antibody (Abcam). Secondary detection was performed using goat anti-mouse IgG antibody conjugated to Cy3 (Jackson ImmunoResearch, Pennsylvania, USA). The DNA was denatured with 2 M HCL and neutralized with 2 M sodium borate buffer (pH 8.5). Nuclei were counterstained with DAPI (Thermo Fisher Scientific). The cells were permeabilized with 0.5% Triton X-100 in PBS at room temperature for 10 min, followed by three PBS washes. The experiments were performed using an inverted fluorescence microscope (Olympus BX51; Olympus Corporation, Tokyo, Japan).

### Plasmid construction and luciferase reporter assay

A DNA sequence ranging from −2000 bp to 500 bp relative to the promoter region of HMGB3 was inserted into the pGL3-Basic vector to construct a reporter plasmid. This plasmid was used as a template, and site-directed mutagenesis was performed to alter the predicted binding site (−944 bp to −933 bp) from 5′-CAACACCTGCCA-3′ to 5′-TCCTCTTGATTC-3′. DNA sequencing confirmed modifications in the promoter fragments. Luciferase activity was analyzed using a dual-luciferase kit (Promega). Transfected cells were lysed and centrifuged at 13000 rpm for 1 min. Relative luciferase activity was assessed using a Modulus TD20/20 luminometer (Turner Biosystems).

### Xenograft and metastasis experiment *in vivo*

Six-week-old BALB/c nude mice were obtained from the Animal Center of the Fourth Military Medical University with prior approval from the Institutional Animal Ethics Committee (approval No. 20230151). All procedures strictly adhered to the guidelines of the Animal Research and Care Committee of the Fourth Military Medical University and were performed under specific pathogen-free conditions. Randomization was performed using a computer-generated number table to allocate mice to the experimental and control groups before the study began. Throughout the study, personnel responsible for outcome assessment and data analysis remained blind to group allocation. The xenograft tumor model was established as described previously.^34^ Each mouse was subcutaneously injected with 5 × 10^6^ cells in the left flank. Every 3 days, both tumor size and weight were assessed, and tumor volume was determined using the following formula: volume = (length × width^2^)/2. Mice were sacrificed after the largest tumor in any group approached 1000 mm^3^. The tumors were embedded in paraffin, sectioned, and fixed on slides. IHC was performed using anti-Kiel-67 (Ki67) (1:1000, Abcam), anti- HMGB3 antibody (1:800, Abcam), or anti-TGIF2 antibody (1:500, Abcam). For *in vivo* metastasis experiments, each mouse was injected with 4 × 10^6^ cells in the tail vein. The mice were euthanized after 6–8 weeks because of declining health, and their livers and lungs were harvested for statistical analysis and histological staining. In addition, the survival duration of mice was documented.

### IHC

ESCC tissue microarray (TMA) slides, acquired from Outdo Biotech Co., Ltd. (Shanghai, China), included 114 ESCC samples and 66 paratumor tissues. However, two ESCC samples and eight paratumor tissues were incomplete and subsequently excluded from the analysis. The final dataset comprised 112 ESCC samples and 57 paratumor tissues. Tissue samples were collected from patients who underwent radical esophagectomy between April 2006 and December 2008. The TMA slides were deparaffinized using a series of xylene and graded alcohol. Endogenous peroxidase activity was inhibited with 3% H_2_O_2_, and non-specific binding was blocked using 10% goat plasma. The TMA slides were incubated with an anti-HMGB3 (1:800, Abcam), anti-TGIF2 (1:500, Abcam), anti-TLR3 (1:500, Abcam), or anti-TGF-β (1:500, Abcam) antibody at 4 °C overnight. Slides were subsequently incubated with biotinylated secondary antibodies and streptavidin-conjugated horseradish peroxidase. The slides were stained with DAB for visualization, followed by hematoxylin counterstaining, and scanned using a panoramic digital slide scanner (3DHISTECH; Sysmex, Hungary). Images were analyzed using the CaseViewer software (3DHISTECH; Sysmex). IHC was used to assess TLR3 expression in 20 pairs of ESCC and adjacent paratumor tissues. Tissues were treated with an anti-TLR3 antibody (1:500; Abcam).

The staining intensity and stained cell percentage on TMA slides were independently evaluated by two pathologists using a histochemical score (H-score). Staining intensity was assessed on a scale of 0–3 as follows: 3 (strong staining), 2 (moderate staining), 1 (weak staining), or 0 (no staining). The area of stained cells was categorized as follows: 0 (0%, no staining), 1 (1%–25% staining), 2 (26%–50% staining), 3 (51%–75% staining), or 4 (76%–100% staining). The H-score was determined by multiplying the staining intensity by the area of stained cells.

### Co-immunoprecipitation (Co-IP) assay

A non-denaturing lysis buffer with an IP kit (ab206996, Abcam) was used to extract total proteins from ECA109 and EC9706 cells. The proteins were incubated with anti-mouse IgG (Beyotime Biotechnology; Shanghai, China) and protein A/G Sepharose for 30 min, followed by the removal of Sepharose. Subsequently, the proteins were co-immunoprecipitated with 5 μg of an anti-rabbit IgG (Beyotime Biotechnology) or primary antibodies (HMGB3, Abcam, ab75782; TLR3, Novus Biologicals, NBP-24875; His-tag, protein-tech, 66005-1-Ig). Next, A/G Sepharose was mixed with the immune complexes and incubated overnight at 4 °C, collected by centrifugation, and washed thrice with lysis buffer. Next, one-quarter of the loading buffer (Beyotime Biotechnology) was mixed with the samples, and the samples were boiled for 10 min before being subjected to WB.

### Molecular docking between HMGB3 and TLR3

The protein–protein docking between HMGB3 and TLR3 was performed using the HDOCK server. The initial sequences were retrieved from UniProtKB (UniProtID: HMGB3, O15347; TLR3, O15455), and their three-dimensional structures were predicted using SWISS-MODEL, with the highest-quality models selected as input. Analysis of the top 10 docking poses revealed a top-ranked model with a docking score of −254.87 and a confidence score of 0.8907. This result suggests potential complex stability, as lower HDOCK scores correlate with stronger binding affinities and greater complex stability. The predicted binding interface residues are detailed in Table S4. Based on these predictions, we generated expression plasmids carrying alanine-scanning mutations targeting three key residues (GLU-576, THR-710, VAL-720) on TLR3 and engineered a His tag, and subsequently performed Co-IP to validate the HMGB3-TLR3 interaction and elucidate its binding mechanism.

### Chromatin immunoprecipitation (ChIP) assay

The physical binding of the HMGB3 promoter to TGIF2, the TGF-β promoter to P65, and the TLR3 promoter to P65 was identified using the ChIP assay. The indicated esophageal cancer cells (3 × 10^6^) were used for ChIP assays using a Magna ChIP G assay kit (Millipore). The cell lines were crosslinked with 1% formaldehyde at 37 °C for 10 min. After quenching with glycine, the extracted bound DNA was coimmunoprecipitated with primary antibodies against normal IgG (Cell Signaling Technology), TGIF2 (Santa Cruz, sc-390870), or P65 (#8242S, Cell Signaling Technology). qRT-PCR was performed to analyze the DNA fragments. The primers used are listed in Table S2.

### Detection of secreted TGF-β

Culture media from EC9706 cells infected with LV-HMGB3 and LV-control or ECA109 cells transfected with si-HMGB3-1, si-HMGB3-2, and si-control were collected after 24 h of incubation. TGF-β levels in media were quantified using the human TGF-β1 ELISA kit (Proteintech; Illinois, USA). A TGF-β-neutralizing antibody 1D11 (Selleck; HOU, USA, 10 μg/mL) was utilized to block the secretion of HMGB3 in the culture medium. Subsequent *in vitro* analysis was conducted to identify the impact of secreted TGF-β in ESCC.

### Flow cytometry

To assess TLR3 subcellular localization, ECA109 cells were stained with an anti-TLR3 antibody (Santa Cruz, Cat. sc-32232; dilution 1:20) at 4 °C for 60 min under either permeabilized or non-permeabilized conditions. Flow cytometry was subsequently performed using a flow cytometer to quantify staining signals.

### Bioinformatic analysis

The expression of HMGB3 in esophageal carcinoma (ESCA) and its correlation with patient survival were analyzed using the Gene Expression Profiling Interactive Analysis 2 (GEPIA2) web server (http://gepia2.cancer-pku.cn/). The ESCA cohort from TCGA was selected. For the survival analysis, patients were stratified into “High” and “Low” expression groups based on the median expression value, and a log-rank test was performed. For the differential expression analysis, the thresholds were set as *P*-value <0.05. Normal tissue data from TCGA were used as the control group.

### Statistical analysis

All statistical analyses were performed with IBM SPSS Statistics (version 22.0). Continuous variables were expressed as mean ± standard deviation. Group comparisons were made using the two-tailed Student’s *t*-test (for two groups) or one-way ANOVA (for multiple groups), with appropriate non-parametric alternatives (the Mann–Whitney *U* test or Kruskal–Wallis test) applied if normality assumptions were violated. The chi-squared test was employed to assess the association between the expression of molecules and clinicopathological parameters of patients with ESCC. Independent factors impacting the survival of patients were identified using the Cox proportional hazards model. The survival rates of patients were evaluated using the log-rank test and the Kaplan–Meier method. Differences between the variables in the groups were examined using a two-tailed Student’s *t*-test. The significance level of *P* < 0.05 was established.

## Results

### Overexpression of HMGB3 promotes proliferation and metastasis in ESCC and indicates a poor prognosis

First, we evaluated HMGB3 expression using the GEPIA database (http://gepia.cancer-pku.cn/) to explore its function in ESCC. HMGB3 expression was notably increased in ESCA tissues than in paratumor tissues (Fig. S2A), which was validated in 20 pairs of ESCC and paratumor tissues using qRT-PCR (*P* = 0.007) (Fig. 1A). Moreover, HMGB3 mRNA and protein levels were markedly elevated in ESCC cell lines than in Het-1A cells, a human immortal esophageal squamous epithelial cell line (Fig. 1B and C). Survival analysis results from the GEPIA database revealed that increased HMGB3 expression was associated with a decreased overall survival (OS) rate (Fig. S2B). Subsequently, IHC was used for protein expression analysis to evaluate the relationship between clinical characteristics and HMGB3 expression. HMGB3 protein levels were significantly higher in ESCC tissues than in paratumor tissues (*P* < 0.001) (Fig. 1D and E; Fig. S2C). Increased HMGB3 protein expression was significantly associated with an advanced American Joint Committee on Cancer (AJCC) stage (*P* = 0.007) and a higher probability of tumor invasion grade (*P* = 0.05). Furthermore, males showed elevated expression of HMGB3 (*P* = 0.046) (Table 1). Kaplan–Meier analysis revealed that individuals with lower HMGB3 expression had longer OS than did those with higher HMGB3 expression (Fig. 1F). Overall, HMGB3 is instrumental in ESCC development.

**Figure 1:**
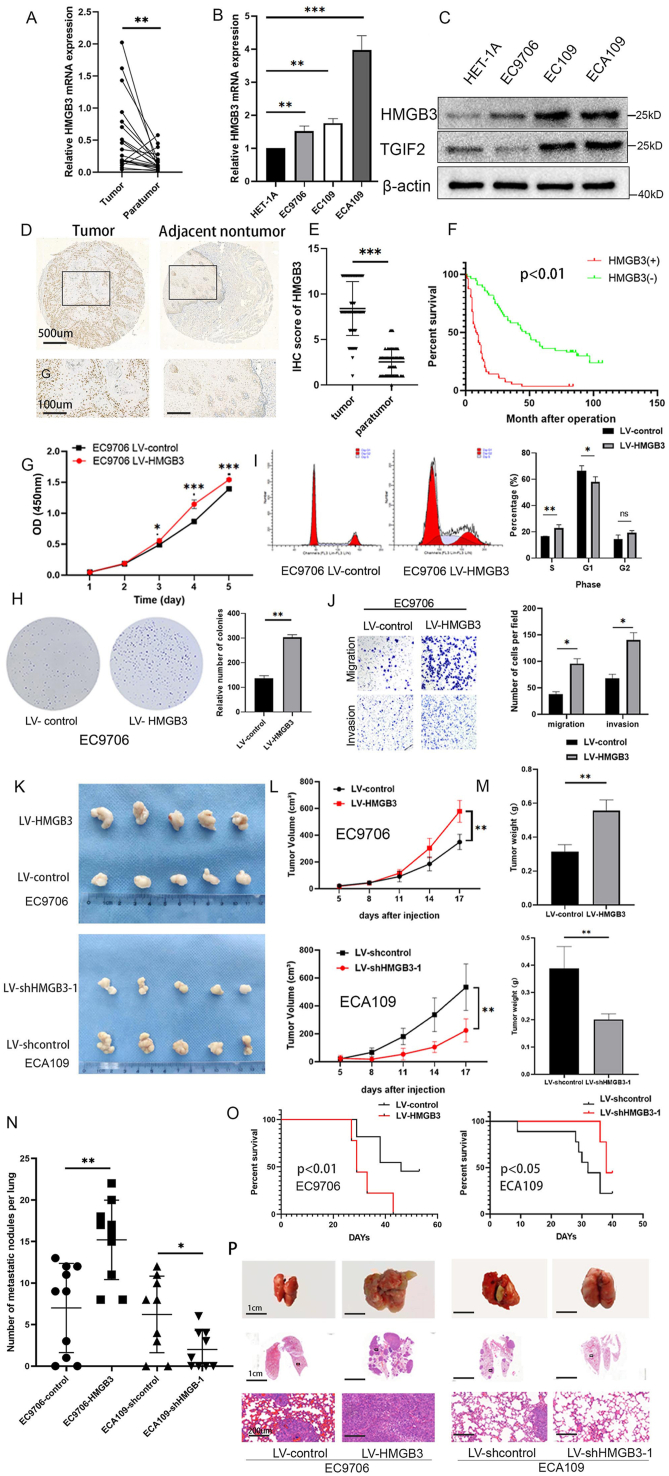
Overexpression of HMGB3 promotes proliferation and metastasis in esophageal squamous cell carcinoma (ESCC) and indicates a poor prognosis. **(A)** Quantitative PCR analysis indicates the expression of HMGB3 in 20 pairs of ESCC tissues and their corresponding paratumor tissues. **(B)** Quantitative PCR analysis was utilized to determine the relative mRNA expression of HMGB3 in ESCC cells. **(C)** Western blotting was performed to evaluate the HMGB3 and TGIF2 protein levels in ESCC cells. **(D)** Representative immunohistochemistry images for HMGB3 staining in ESCC and corresponding paratumor tissues. **(E)** Semi-quantitative analysis was utilized to assess the immunohistochemistry scores of HMGB3 in both ESCC and their corresponding paratumor tissues (*n* = 57 pairs). **(F)** Kaplan–Meier analysis demonstrates the association between the mRNA expression of HMGB3 and overall survival in ESCC patients (*n* = 112). The red line indicates patients with relatively higher HMGB3, whereas the green line indicates the group with lower HMGB3. **(G)** CCK-8 assay demonstrates the alteration in the proliferative ability of ESCC cells upon HMGB3 overexpression, with optical density (OD) measurements recorded daily for 5 days. **(H)** Colony formation assay illustrates the proliferative ability of ESCC cells following the overexpression of HMGB3. The left panel depicts the results, whereas the right panel illustrates the number of counted colonies within the designated groups. **(I)** The impact of HMGB3 overexpression on the cell cycle was evaluated by flow cytometry (left), with percentages of cells in the S, G1, and G2 phases determined and compared (right). **(J)** Transwell analysis demonstrates the migratory and invasive capacity of ESCC cells following the overexpression of HMGB3 (left). The numbers of migrated and invasive cells were quantified and compared (right). **(K**–**M)** Nude mice, categorized into four groups, each consisting of five mice, were injected with the designated cells. (K) Images depicting the xenograft tumors. (L, M) Tumor volume and weight data from xenograft mouse tumors. **(N–P)** Nude mice, categorized into four groups, each consisting of 10 mice, were administered the specified cells via subcutaneous injection through the tail vein. (N) The number of metastatic nodules in the lungs was determined for each group. (O) Overall survival of the nude mice in the indicated four groups. (P) Representative hematoxylin-eosin staining images of lung tissues from the specified groups are presented. All data were expressed as mean ± standard deviation. Statistical significance is indicated as ∗*P* < 0.05, ∗∗*P* < 0.01, and ∗∗∗*P* < 0.001.

**Table 1:** Association of HMGB3, TGIF2, and TGF-β expression with clinicopathological features in an independent cohort of esophageal squamous cell carcinoma tissue.

Clinicopathological variables	HMGB3		*P* value	TGIF2		*P* value	TGF-β		*P* value
	Low	High		Low	High		Low	High	
Age			0.26			0.076			0.464
≤65	29	24		22	31		26	27	
>65	26	33		33	25		30	28	
Sex			**0.046**			0.414			**0.042**
Female	18	10		15	13		19	10	
Male	37	47		41	43		37	46	
Pathological grade			0.369			**0.006**			**0.037**
Ⅰ	2	5		1	6		1	7	
Ⅱ	40	42		37	44		40	40	
Ⅲ	13	10		18	6		15	9	
Tumor size (cm)			0.161			0.079			0.116
≤5	40	34		40	33		39	33	
>5	13	20		12	21		13	20	
Tumor invasion			0.05			0.067			0.168
T1	4	1		2	3		3	2	
T2	13	5		14	4		12	5	
T3	36	47		38	44		37	46	
T4	0	1		0	1		1	0	
Lymph node metastasis			0.074			0.705			0.224
Absent	31	22		27	25		28	23	
Present	24	35		29	31		28	33	
American joint committee on cancer stage			**0.007**			0.12			0.059
Stage Ⅰ	4	1		2	3		3	2	
Stage Ⅱ	30	18		29	18		28	18	
Stage Ⅲ	19	36		23	32		23	33	

siRNA was transfected into EC109, ECA109, and primary ESCC cells to silence HMGB3 expression. EC9706, KYSE-150, and primary ESCC cells were used to establish HMGB3 overexpression cell models by infecting cells with a lentiviral vector. The qRT-PCR results (Fig. S2D and S2E) and WB (Fig. S2F) validated HMGB3 expression. The CCK-8 assay results (Fig. 1G; Fig. S2G) and colony formation assays (Fig. 1H; Fig. S2H) demonstrated that HMGB3 down-regulation diminished the proliferative capabilities of ESCC cells, whereas its up-regulation enhanced these abilities. Furthermore, HMGB3 depletion in EC109 and ECA109 cells caused cell cycle arrest. A significantly lower number of cells were in the S phase, whereas more cells were in the G1 phase compared with cells transfected with siRNA-NC (Fig. S2I). HMGB3 overexpression induced the accumulation of cells in the S phase in EC9706 cells (Fig. 1I). Consistently, BrdU incorporation experiments demonstrated that HMGB3 overexpression increased the number of EC9706 cells in the S phase (Fig. S2J). The Transwell assay confirmed that HMGB3 knockdown suppressed the invasion and migration of EC9706 cells. In contrast, increased HMGB3 expression stimulated the invasion and migration of ESCC cells (Fig. 1J; Fig. S2K).

Stable cell lines were generated using ECA109 cells by infection with lentivirus-control, lentivirus-shHMGB3-1, and lentivirus-shHMGB3-2. The qRT-PCR and WB results indicated that LV-shHMGB3-1 infection effectively decreased the mRNA and protein expression of HMGB3 in ECA109 cells (Fig. S2L, S2M). Tumor volume and weight were reduced in the LV-shHMGB3-1 group compared with those in the LV-shcontrol group, whereas HMGB3 overexpression facilitated tumor growth *in vivo* (Fig. 1K–M). The Ki67 level was lower in HMGB3-down-regulated ECA109 cells than in the NC group, whereas the Ki67 level increased in EC9706 cells infected with LV-HMGB3 compared with the NC group (Fig. S2N, S2O). Additionally, we conducted *in vivo* metastasis experiments, indicating that HMGB3 overexpression reduced the OS of mice in the HMGB3-up-regulated group and increased the incidence of lung metastasis in ESCC cells, whereas HMGB3 knockdown exerted opposite effects (Fig. 1N and O). Hematoxylin-eosin staining revealed that the HMGB3 overexpression group had more metastatic lung nodules, whereas HMGB3 knockdown decreased the metastatic potential of ESCC cells *in vivo* (Fig. 1P). Thus, HMGB3 contributes to oncogenic activity by promoting ESCC cell proliferation and metastasis, as was evident from both *in vitro* and *in vivo* experiments.

### TGIF2 regulates HMGB3 transcriptionally by binding to its promoter

To explore the potential mechanisms underlying HMGB3 up-regulation, we used the JASPAR database (http://jaspar.genereg.net/) combined with the UCSC database (http://genome.ucsc.edu/) to predict the upstream transcription factors that directly bind to the HMGB3 promoter (Fig. S3A). TGIF2 is a notable oncogene because of its involvement in the development and progression of different carcinomas (Fig. S3A). As depicted in Fig. S3B and a positive association between HMGB3 and TGIF2 expression in ESCC tissues was observed in the GEPIA database (*R* = 0.18, *P* = 0.018). Subsequently, qRT-PCR (Fig. S3C) and WB (Fig. 1C) demonstrated that TGIF2 expression was evaluated in Het-1A and ESCC cell lines. TGIF2 expression was reduced in EC9706 cells compared with that in Het-1A cells, whereas it was elevated in EC109 and ECA109 cells. Additionally, qRT-PCR and WB were performed to assess the regulatory role of TGIF2 in HMGB3 expression in ESCC. HMGB3 expression was down-regulated upon TGIF2 silencing, whereas its level was up-regulated upon TGIF2 overexpression in ESCC cells (Fig. S3D–S3G). Finally, ChIP and luciferase reporter assays were conducted to precisely validate the binding site of TGIF2 in the promoter sequence of HMGB3. The HMGB3 promoter sequence was found to be enriched in anti-TGIF2 immunoprecipitates compared with normal IgG immunoprecipitates of cell lysates (Fig. 2A–C). Both the ChIP assay and luciferase reporter experiment confirmed that the region between −894 bp and −1138 bp (denoted as ChIP 2 region) within the HMGB3 promoter serves as a specific binding site for TGIF2, with a distal control region (ChIP NC) included to verify binding specificity. Building on previous studies showing that EGFR signaling drives TGIF2 phosphorylation to potentiate the transcriptional activation of downstream genes,^23^ we hypothesized that HMGB3 expression may also be regulated by phosphorylated TGIF2. Consistent with this hypothesis, WB analysis demonstrated that EGF-mediated EGFR activation induced TGIF2 phosphorylation, followed by a marked increase in HMGB3 protein levels (Fig. S3H). This regulatory cascade was abolished by pre-treatment with gefitinib, an EGFR inhibitor, confirming EGFR signaling dependency (Fig. S3I). Altogether, there exists a possible mechanistic link between EGFR signaling-mediated TGIF2 phosphorylation and transcriptional activation of HMGB3.

**Figure 2:**
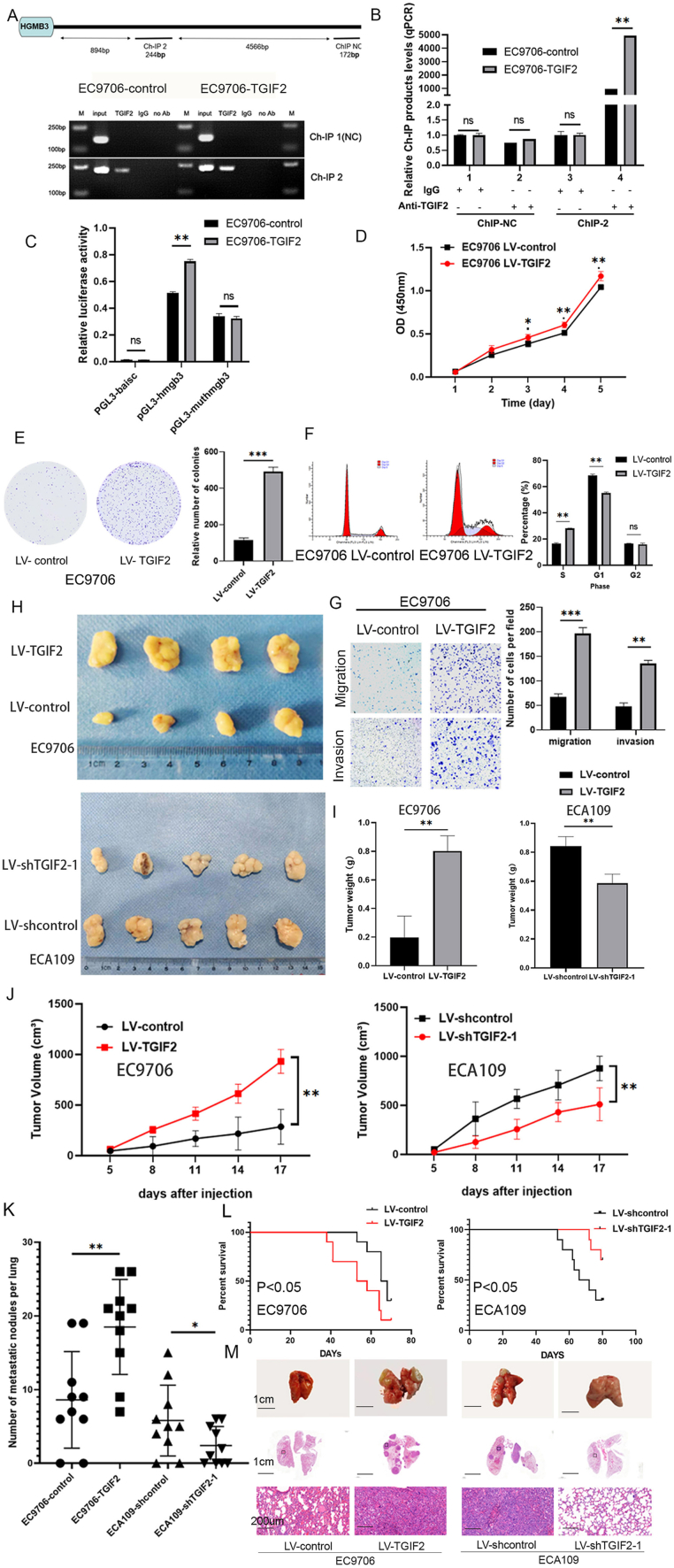
TGIF2 regulates HMGB3 transcriptionally by binding to its promoter and promotes proliferation and metastasis of esophageal squamous cell carcinoma (ESCC). **(A, B)** Chromatin immunoprecipitation and quantitative PCR assays demonstrated that the HMGB3 promoter could be directly bound by TGIF2, with the binding site located from −1138 bp to −894 bp. **(C)** EC9706 cells infected with LV-control or LV-TGIF2 were transfected with serially truncated or mutated HMGB3 promoter constructs, which were cloned into the pGL3-luciferase reporter plasmid. Subsequently, relative luciferase activity was measured. **(D)** The proliferative capacity of ESCC cells following TGIF2 overexpression was evaluated using the CCK-8 assay, with optical density (OD) measurements recorded daily for 5 days. **(E)** The proliferative capacity of ESCC cells was evaluated after TGIF2 overexpression (left) using a colony formation assay. The number of colonies within the designated groups was quantified and compared (right panel). **(F)** Flow cytometry was used to evaluate the effect of TGIF2 up-regulation on cell cycle distribution (left). The percentages of cells in the G1, G2, and S phases were computed and analyzed (right). **(G)** The migratory and invasive abilities of ESCC cells following TGIF2 up-regulation were demonstrated via Transwell assay (left). The numbers of migrated and invasive cells were calculated and compared (right). **(H**–**J)** Nude mice, categorized into four groups, each consisting of four or five mice, were subcutaneously injected with the designated cells. (H) Images depicting xenograft tumors. (I, J) Data regarding the tumor volumes and weights. **(K**–**M)** Nude mice were categorized into four groups, each consisting of 10 mice, and were administered the specified cells via subcutaneous injection through the tail vein. (K) The number of metastatic nodules in the lungs was calculated and analyzed for each group. (L) Overall survival of nude mice in the four indicated groups. (M) Representative hematoxylin-eosin-stained images of lung samples from specific groups are presented. All data are expressed as mean ± standard deviation. Statistical significance is indicated by ∗*P* < 0.05, ∗∗*P* < 0.01, and ∗∗∗*P* < 0.001.

### Elevated TGIF2 expression promotes proliferation, migration, and invasion of ESCC cells *in vitro* and *in vivo*

We used the cell lines in which TGIF2 was either overexpressed or knocked down to perform the CCK-8 assay (Fig. 2D; Fig. S3J). Additionally, we conducted colony formation experiments to investigate the effect of TGIF2 on ESCC cell proliferation (Fig. 2E; Fig. S3K). Increasing TGIF2 levels boosted ESCC cell proliferation, whereas reducing TGIF2 levels delayed these harmful effects. A cell cycle assay was performed to verify the effect of TGIF2 on cell cycle progression (Fig. 2F; Fig. S3L). The number of cells in the S phase decreased in ECA109 cells infected with LV-shTGIF2, whereas that in the G1 phase increased. Conversely, more cells were in the S phase, and fewer cells were in the G1 phase after EC9706 cells were infected with LV-TGIF2. Alternatively, the BrdU incorporation experiment corroborated that TGIF2 overexpression in EC9706 cells promoted S phase progression (Fig. S3M). As depicted in Figure 2H–J, tumor volumes and weights were notably greater in the TGIF2 overexpression group compared with those in the NC group. Conversely, tumors generated from TGIF2-deleted ESCC cells displayed markedly reduced volumes and weights compared with those generated from the NC group. Reduced Ki67 staining was observed in TGIF2-down-regulated ECA109 cells, and *vice versa* (Fig. S3N, S3O). Furthermore, TGIF2 overexpression augmented the invasive and metastatic capabilities of ESCC cells, whereas its knockdown had the opposite effect (Fig. 2G; Fig. S3P). Elevated TGIF2 levels reduced the OS in mice and correlated with an increased incidence of metastatic lung nodules (Fig. 2K–M). In summary, TGIF2 drives ESCC cell proliferation and metastasis and functions as an oncogene both *in vitro* and *in vivo*. Furthermore, we established a patient-derived organoid model of ESCC and employed siRNAs to knock down TGIF2 and HMGB3. Subsequent cell proliferation assays using CTG demonstrated that depletion of TGIF2 and HMGB3 significantly suppressed ESCC organoid growth, suggesting their critical roles in tumor progression (Fig. S3Q, S3R).

### HMGB3 plays a crucial role in facilitating TGIF2-induced cell proliferation and metastasis in ESCC cells

**Figure 3:**
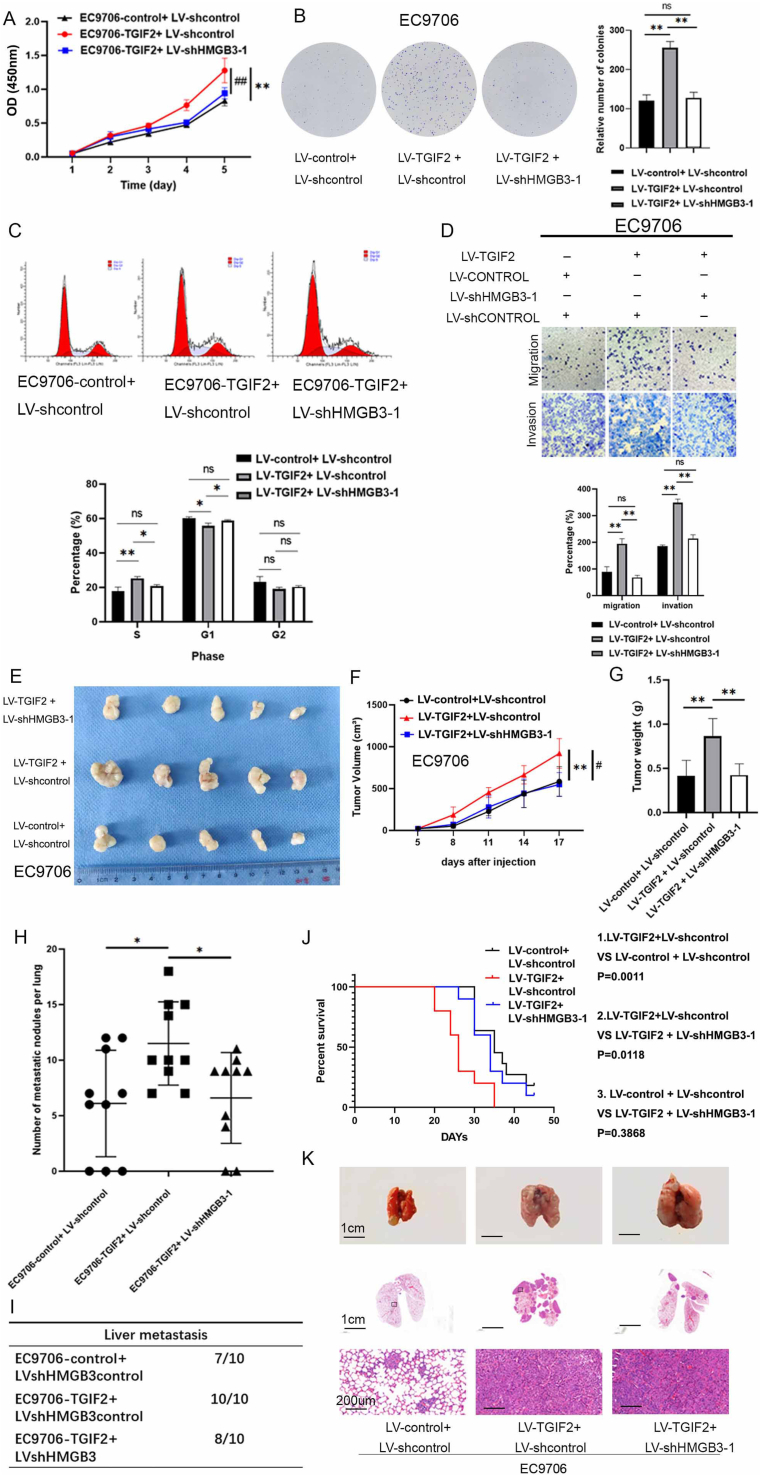
HMGB3 plays a crucial role in facilitating TGIF2-induced cell proliferation and metastasis in esophageal squamous cell carcinoma (ESCC) cells. **(A)** The proliferative ability of ESCC cells following the expression modulation of HMGB3 and TGIF2 was assessed using the CCK-8 assay. Optical density (OD) measurements were obtained daily for 5 days (“∗” denotes a comparison between LV-TGIF2 plus LV-shcontrol and LV-control plus LV-shcontrol, whereas “^#^” signifies a comparison between LV-TGIF2 plus LV-shHMGB3-1 and LV-TGIF2 plus LV-shcontrol). **(B)** The proliferative ability of ESCC cells subsequent to altering the expression of TGIF2 and HMGB3 was demonstrated by the colony formation assay (left). The colony number in each group was quantified and compared (right). **(C)** The impact of TGIF2 on the cell cycle is partially dependent on HMGB3 (up), as revealed by the flow cytometry analysis. The percentages of cells in the G1, G2, and S phases were analyzed (down). **(D)** Transwell analysis demonstrates the migratory and invasive abilities of ESCC cells following modulation of TGIF2 and HMGB3 expression (up). Subsequently, the numbers of migrated and invasive cells were quantified and compared (down). **(E**–**G)** Nude mice, categorized into four groups, each consisting of five mice, were subcutaneously injected with the designated cells. (E) Images depicting the xenograft tumors. (F, G) Data regarding the tumor volumes and weights of the xenograft tumors. **(H–K)** Nude mice, categorized into four groups, each consisting of 10 mice, were administered the specified cells via subcutaneous injection through the tail vein. (H, I) The number of metastatic nodules in the lungs was calculated and analyzed for each group. (J) Overall survival of the nude mice in the indicated three groups. (K) Images depicting representative hematoxylin-eosin staining of lung samples from the specified groups. All data were expressed as mean ± standard deviation. Statistical significance is indicated as ∗*P* < 0.05, ∗∗*P* < 0.01, and ∗∗∗*P* < 0.001.

**Figure S4:**
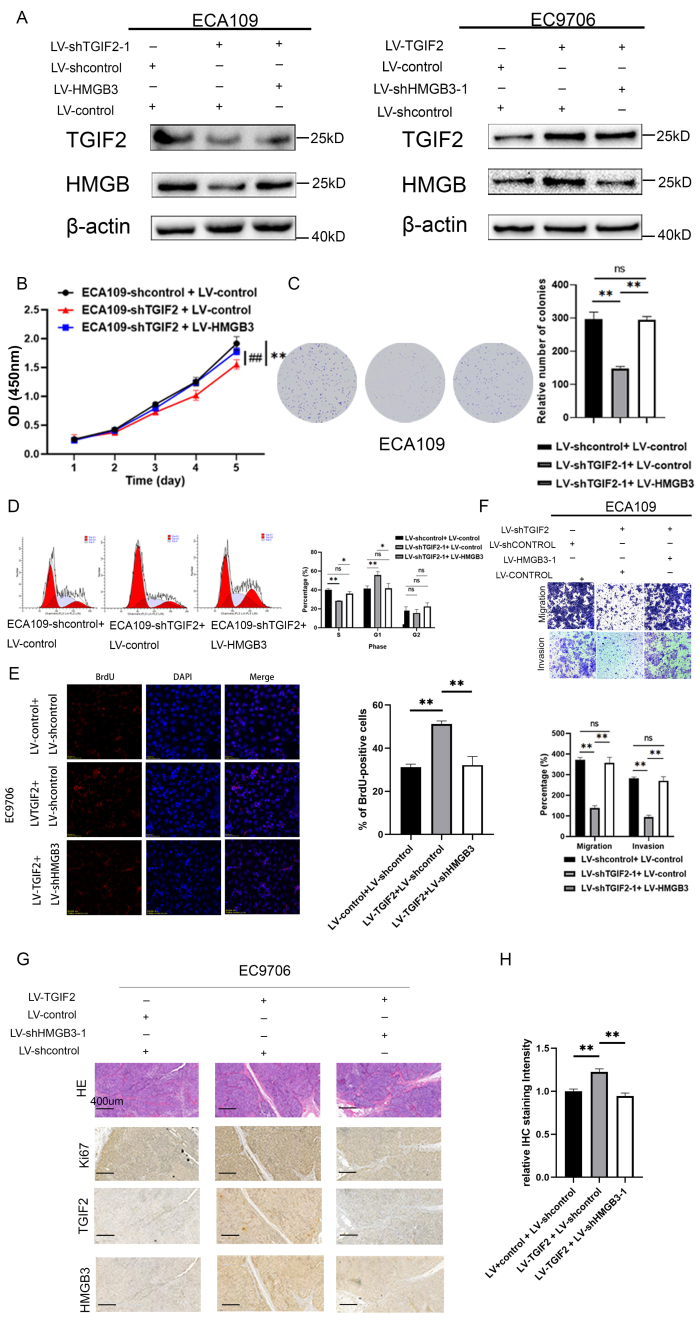
HMGB3 plays a crucial role in facilitating TGIF2-induced cell proliferation and metastasis in esophageal squamous cell carcinoma (ESCC) cells. **(A)** Protein expression of TGIF2 in ECA109 and EC9706 cells was analyzed by Western blotting following lentivirus infection. **(B)** The CCK-8 assay indicates significant changes in the proliferation of ESCC cells following the modulation of HMGB3 and TGIF2 expression, with optical density (OD) detected daily for 5 days. In ECA109 cells, “∗” indicates a comparison between LV-shTGIF2 plus LV-control and LV-shcontrol plus LV-control, whereas “^#^” indicates a comparison between LV-shTGIF2 plus LV-HMGB3 and LV-shTGIF2 plus LV-control. **(C)** The colony formation assay demonstrates the proliferative capability of ESCC cells following the modification of TGIF2 and HMGB3 expression (left). The number of colonies was counted and compared among the indicated groups (right). **(D)** The flow cytometry analysis demonstrates that TGIF2’s impact on the cell cycle is partially dependent on HMGB3 (top). The percentage of cells in the G1, G2, and S phases of the cell cycle was calculated and compared accordingly (bottom). **(E)** The BrdU incorporation assay demonstrates that TGIF2’s impact on the S phase of the cell cycle (left), with the relative BrdU incorporation levels being quantified and compared accordingly (right). **(F)** The Transwell analysis reveals the migratory and invasive capabilities of ESCC cells after modifying the expression of TGIF2 and HMGB3 (top). The numbers of migrated and invasive cells were quantified and compared (bottom). **(G)** Representative images of hematoxylin-eosin and immunohistochemistry staining for HMGB3, TGIF2, and Ki67 in tumor tissues from xenograft mouse models. All data were expressed as mean ± standard deviation. **(H)** Ki67 expression levels were quantified, and the relative Ki67 levels were calculated. Statistical significance is denoted as ∗∗∗*P* < 0.001, ∗∗*P* < 0.01, and ∗*P* < 0.05. ^#^*P* < 0.05, ^##^*P* < 0.01 and ^###^*P* < 0.001.

To examine the role of HMGB3 in TGIF2-induced proliferation and metastasis of ESCC cells, TGIF2-overexpressing EC9706 cells were infected with LV-shHMGB3-1, and TGIF2-knockdown ECA109 cells were infected with HMGB3 overexpression lentivirus. TGIF2 and HMGB3 expressions were confirmed by WB (Fig. S4A). The CCK-8 and colony formation assays demonstrated that the proliferation and colony formation abilities of ESCC cells were significantly inhibited by HMGB3 silencing in TGIF2-overexpressing EC9706 cells (Fig. 3A and B; Fig. S4B and S4C). Conversely, HMGB3 overexpression partially reversed the TGIF2 knockdown-induced suppressive effects of ESCC cell proliferation. Furthermore, the cell cycle assay revealed that HMGB3 knockdown in TGIF2-overexpressing EC9706 cells decreased the number of cells in the S phase and increased in the G1 phase (Fig. 3C; Fig. S4D). Similarly, BrdU incorporation assays revealed that HMGB3 knockdown in TGIF2-overexpressing EC9706 cells significantly attenuated S phase progression (Fig. S4E). HMGB3 down-regulation in TGIF2-overexpressing EC9706 cells markedly reduced the volume and weight of xenograft tumors (Fig. 3E–G). The Ki67 staining was diminished following HMGB3 knockdown in TGIF2-overexpressing EC9706 cells (Fig. S4G and S4H)**.** Furthermore, Transwell assays confirmed that HMGB3 down-regulation in TGIF2-overexpressing EC9706 cells reduced their invasion and migration rate, whereas HMGB3 up-regulation in TGIF2-knockdown ECA109 cells enhanced their capabilities (Fig. 3D; Fig. S4F). In the *in vivo* metastatic experiment, HMGB3 knockdown in TGIF2-up-regulated EC9706 cells resulted in elevated OS rate, decreased incidence of lung metastasis, and fewer metastatic lung nodules in nude mice (Fig. 3H–K). Therefore, HMGB3 facilitates TGIF2-induced proliferation and metastasis of ESCC cells.

### HMGB3 and TGIF2 activate TGF-β signaling in ESCC

**Figure 4:**
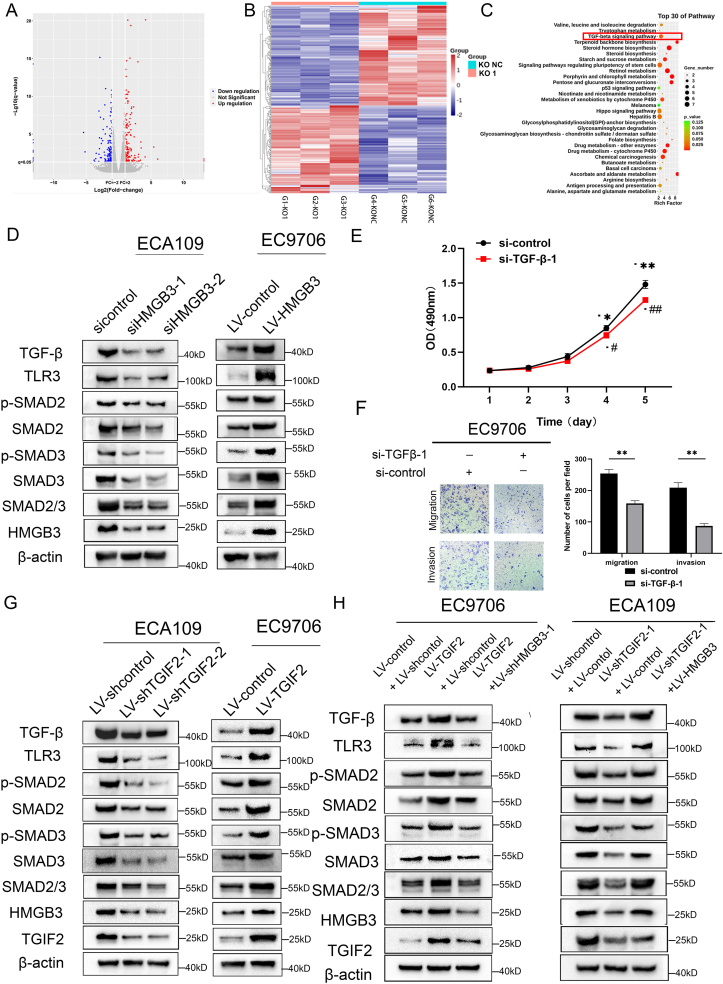
HMGB3 and TGIF2 activate TGF-β signaling in esophageal squamous cell carcinoma (ESCC). **(A)** RNA sequencing analysis was performed on ECA109 cells infected with LV-control and LV-shHMGB3-1. The RNA sequencing volcano plot highlights 168 genes as up-regulated and 141 genes as down-regulated. Blue dots represent genes with lower expression in ECA109-control compared with ECA109-shHMGB3-1, whereas red dots indicate higher expressed genes. **(B)** The heatmap generated from RNA sequencing data displays 168 up-regulated genes and 141 down-regulated genes when comparing LV-control with LV-shHMGB3-1 in ECA109 cells. Red highlights indicate up-regulated genes, whereas blue highlights indicate down-regulated genes. **(C)** KEGG analysis was used to identify the top 30 most relevant pathways. **(D)** Western blotting analysis demonstrates that the HMGB3 positively regulates Smad-dependent TGF-β signaling. **(E)** The proliferative capacity of ESCC cells after modifying the expression of TGF-β was demonstrated by the CCK-8 assay, with optical density (OD) measurements obtained daily for 5 days. **(F)** The migratory and invasive abilities of ESCC cells after modifying the expression of TGF-β were demonstrated by the Transwell analysis. The left panel illustrates the results, whereas the right panel shows the number of migrated and invasive cells calculated and compared, respectively. **(G)** Western blotting analysis demonstrates that TGIF2 positively regulates Smad-dependent TGF-β signaling. **(H)** WB analysis demonstrates that TGIF2 positively regulates the Smad-dependent TGF-β pathway in an HMGB3-dependent manner. All data were expressed as mean ± standard deviation. Statistical significance is indicated as ∗*P* < 0.05, ∗∗*P* < 0.01, ∗∗∗*P* < 0.001, ^#^*P* < 0.05, ^##^*P* < 0.01, and ^###^*P* < 0.001.

**Figure S5:**
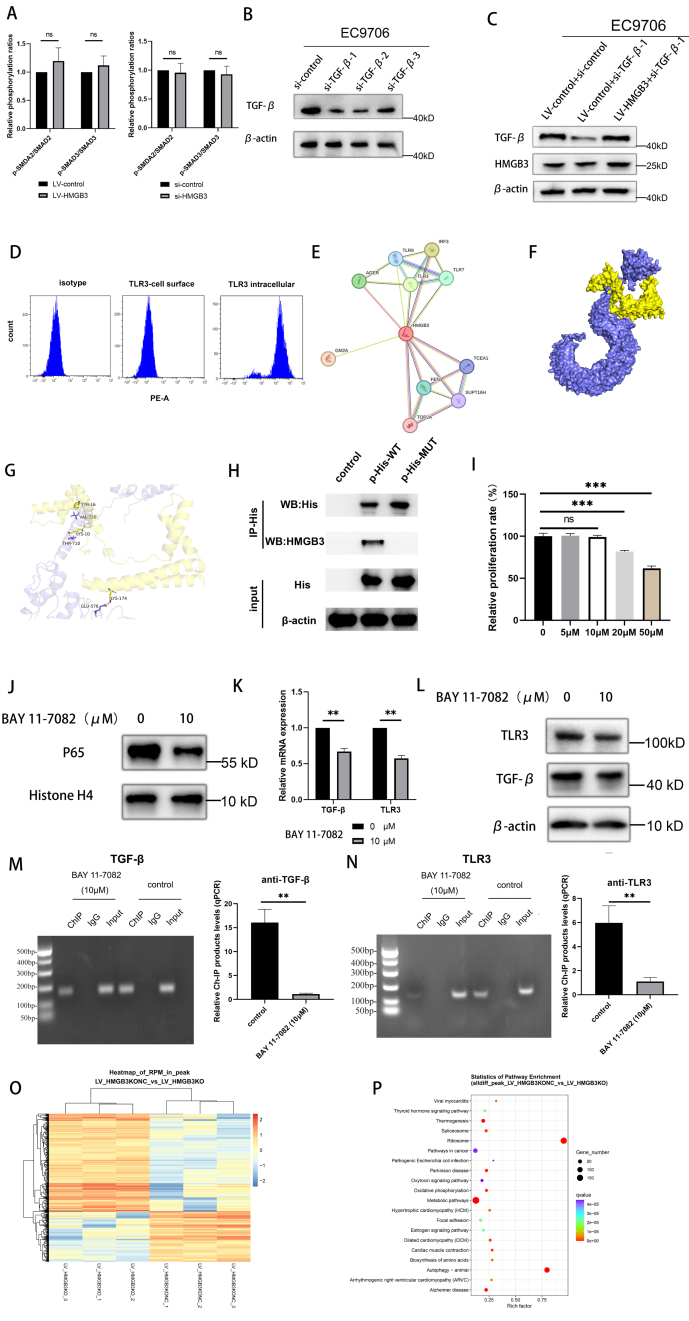
HMGB3 interacts with TLR3 and triggers the Smad-dependent TGF-β signaling pathway via NF-kB signaling in esophageal squamous cell carcinoma. **(A)** Phosphorylation ratios of SMAD2 (p-SMAD2/SMAD2) and SMAD3 (p-SMAD3/SMAD3) were quantified after HMGB3 expression was regulated. **(B)** Relative TGF-β protein expression levels in EC9706 cells following siRNA transfection, as analyzed by Western blotting. **(C)** Relative TGF-β and HMGB3 protein expression levels in EC9706 cells after infection with lentivirus and transfection of siRNA, as analyzed by Western blotting. **(D)** Subcellular localization of TLR3 in ECA109 cells detected by flow cytometry. **(E)** Protein interaction network of HMGB3 predicted by the STRING database analysis. **(F)** Predicted 3D structural model of TLR3-HMGB3 binding from molecular docking. **(G)** Key interaction residues at TLR3–HMGB3 binding interface identified by molecular docking. **(H)** Co-immunoprecipitation analysis of HMGB3–TLR3 interaction using His-tagged wild-type (WT) and binding site mutants (MUT). **(I)** Cytotoxicity assessment of BAY 11–7082 in ECA109 cells by CCK-8 assay. (Dose range: 0, 5, 10, 20, 50 μM). **(J)** Western blotting illustrates the protein levels of nuclear NF-κB P65 after BAY 11–7082 treatment. **(K)** Quantitative PCR demonstrates the RNA levels of TGF-β and TLR3 after NF-κB was inhibited by BAY 11–7082. **(L)** Western blotting analysis illustrates the protein levels of TGF-β and TLR3 after NF-κB was inhibited by BAY 11–7082. **(M, N)** Chromatin immunoprecipitation and quantitative PCR assays demonstrate that enrichment of NF-κB P65 in TGF-β and TLR3 promoter was inhibited by BAY 11–7082. **(O)** ATAC-seq analysis was conducted in ECA109 cells transduced with LV-control or LV-shHMGB3-1. The ATAC-seq heatmap revealed 2148 peaks with increased chromatin accessibility and 1605 genes with decreased accessibility. Blue dots denote regions of reduced chromatin accessibility, while red dots represent regions with higher accessibility. **(P)** KEGG analysis was used to identify the top 20 most relevant pathways.

We conducted RNA sequencing analysis to investigate the potential downstream targets or pathways influenced by HMGB3. Compared with the control group, 168 genes were up-regulated, and 141 genes were down-regulated in ECA109 cells infected with LV-shHMGB3-1 (|fold change| ≥ 2, false discovery rate <0.05) (Fig. 4A and B). Table S5 lists the differentially expressed genes documented. KEGG enrichment analysis revealed that TGF-β signaling activation could potentially represent a downstream pathway regulated by HMGB3 (Fig. 4C). We subsequently conducted WB to understand the potential regulatory impact of HMGB3 on the TGF-β signaling pathway. HMGB3 overexpression increased TGF-β, SMAD2/3, SMAD3, SMAD2, p-SMAD2, and p-SMAD3 expression (Fig. 4D). Conversely, HMGB3 deletion reduced the expression of these molecules. However, quantitative analysis revealed that HMGB3 modulation did not significantly alter the phosphorylation ratios of SMAD2 (p-SMAD2/SMAD2) or SMAD3 (p-SMAD3/SMAD3) (Fig. S5A).

Given that TGF-β is the downstream target of HMGB3, we explored whether HMGB3 enhanced the proliferation and metastasis of ESCC cells by influencing TGF-β signaling. We introduced siRNAs into EC9706 cells to suppress TGF-β expression, and the knockdown efficiency was evaluated using WB (Fig. S5B). We transfected si-TGF-β-1 into both HMGB3-overexpressing EC9706 cells and their corresponding normal controls. Subsequently, HMGB3 and TGF-β expression were quantitatively assessed using WB (Fig. S5C). Subsequent experiments confirmed that TGF-β knockdown inhibited proliferation (Fig. 4E), invasion, and migration (Fig. 4F) of EC9706 cells.

Next, TGIF2 promoted the activation of the TGF-β signaling pathway in a Smad-dependent manner (Fig. 4G). This regulatory effect was partially dependent on HMGB3 expression (Fig. 4H). Additionally, the attenuation of TGF-β signaling activation induced by TGIF2 overexpression in EC9706 cells was observed upon HMGB3 knockdown. Conversely, elevated HMGB3 expression in ECA109 cells reversed the inhibitory impact of TGIF2 knockdown on TGF-β signaling. These findings collectively implicate TGIF2 and HMGB3 in the up-regulation of TGF-β signaling in ESCC, and that TGIF2-induced up-regulation of TGF-β signaling is at least partially dependent on HMGB3.

### HMGB3 interacts with TLR3 and triggers the Smad-dependent TGF-β signaling pathway via NF-kB signaling in ESCC

**Figure 5:**
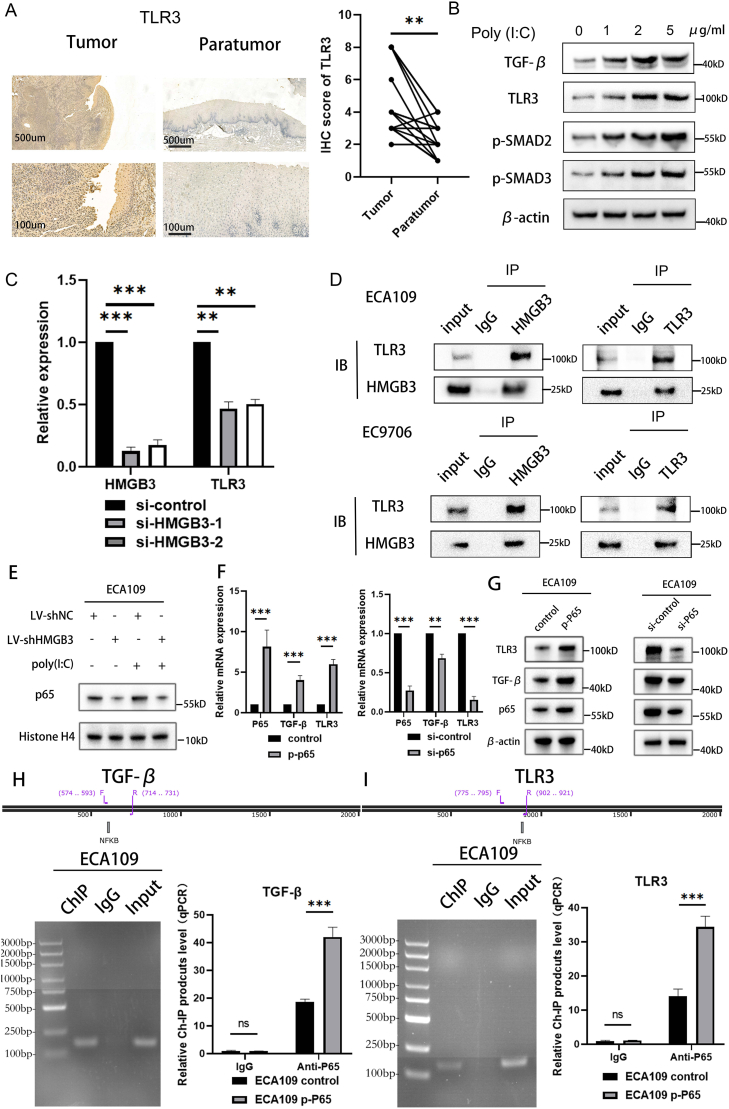
HMGB3 interacts with TLR3 and triggers the Smad-dependent TGF-β signaling pathway via NF-kB signaling in esophageal squamous cell carcinoma (ESCC). **(A)** Immunohistochemistry analysis reveals TLR3 expression in 20 pairs of ESCC tissues and the corresponding paratumor tissues. **(B)** Western blotting analysis of EC9706 cells treated with different concentrations of poly (I:C) reveals that TLR3 positively regulates the Smad-dependent TGF-β pathway. **(C)** Quantitative PCR reveals the RNA expression correlation between HMGB3 and TLR3 in ESCC cell lines. **(D)** The co-immunoprecipitation test was conducted to explore the direct interaction between TLR3 and HMGB3. **(E)** The effect of HMGB3 down-regulation on NF-κB P65 nuclear expression in ECA109 was analyzed by Western blotting. **(F)** Quantitative PCR demonstrates the RNA levels of TGF-β and TLR3 after NF-κB P65 was up-regulated by plasmid or down-regulated by siRNA. **(G)** Western blotting analysis illustrates the protein levels of TGF-β and TLR3 after NF-κB P65 was up-regulated by plasmid or down-regulated by siRNA. **(H, I)** Chromatin immunoprecipitation and quantitative PCR assays demonstrate that TGF-β and TLR3 promoters could be directly bound by NF-κB P65. All data were expressed as mean ± standard deviation. Statistical significance is indicated as ∗*P* < 0.05, ∗∗*P* < 0.01, and ∗∗∗*P* < 0.001.

Recent studies have emphasized the association between HMGB and the TLR family.^29^^,^^35^ As a member of this family, TLR3, which has been reported to be primarily intracellularly localized,^36^^,^^37^ was also confirmed by flow cytometry to localize intracellularly rather than on the cell membrane in ESCC (Fig. S5D). TLR3 is implicated in the spread of the TGF-β signaling pathway in different types of carcinomas.^31^^,^^38^ TLR3 was significantly down-regulated in ECA109 cells infected with LV-shHMGB3-1 compared with the NC group (Table S3). Consequently, we explored the potential association among HMGB3, TLR3, and TGF-β signaling. Initially, TLR3 was overexpressed in ESCC tissues compared with adjacent normal tissues, as determined by IHC (Fig. 5A). TLR3 is stimulated by polyinosinic–polycytidylic acid (poly (I:C)). TLR3 activation elevates the key molecules’ expression within the TGF-β signaling pathway in ESCC (Fig. 5B). Furthermore, TGIF2 or HMGB3 overexpression increased the TLR3 expression, and conversely, TGIF2 or HMGB3 down-regulation decreased the TLR3 expression (Figure 4, Figure 5C). HMGB3 down-regulation reduced TGIF2 overexpression-induced TLR3 and the TGF-β signaling pathway activation in EC9706 cells. HMGB3 up-regulation enhanced TLR3 levels and stimulated TGF-β signaling in ECA109 cells with TGIF2 knockdown, indicating the positive regulatory effect of HMGB3 on TLR3 expression (Fig. 4H). The members of the HMGB and TLR families can function as donors and receptors, respectively, and induce carcinogenesis. A reciprocal Co-IP assay was performed to investigate the association between TLR3 and HMGB3. HMGB3 immunoprecipitation contributes to the detection of endogenous TLR3. Similarly, immunoprecipitation of endogenous TLR3 resulted in the detection of HMGB3 (Fig. 5D). To further validate the HMGB3-TLR3 interaction, we interrogated the STRING database, which indicated a potential interaction between HMGB3 and TLR3 (Fig. S5E). Molecular docking simulations were subsequently performed to predict their possible binding mode and interface residues (Fig. S5F and S5G). Site-directed mutagenesis of the three predicted TLR3 binding sites (GLU-576, THR-710, VAL-720) was then conducted (Table S4). Co-IP assays revealed that mutations at these sites abrogated the HMGB3-TLR3 interaction (Fig. S5H). Collectively, these results demonstrate that HMGB3 might physically interact with TLR3 to activate the Smad-dependent TGF-β signaling pathway in ESCC.

However, the mechanisms underlying TGF-β signaling activation and TLR3 transcriptional regulation following HMGB3–TLR3 interaction remain unclear. Building upon previous findings that TLR family members can activate the NF-κB signaling by interaction with HMGB family members^39^ and considering that P65 is a transcription factor for TGF-β,^40^ we hypothesized a potential regulatory function of NF-κB in this process. This hypothesis was further supported by database mining, which predicted NF-κB as a potential transcriptional regulator of both TGF-β and TLR3 (Fig. 5H and I). We used WB to validate this prediction and assessed the nuclear expression of NF-κB. HMGB3 knockdown reduced the nuclear expression of NF-κB (Fig. 5E). Additionally, poly (I:C)-induced TLR3 overexpression increased NF-κB nuclear expression, which was subsequently inhibited by HMGB3 knockdown (Fig. 5E). To further validate NF-κB’s role in the transcriptional regulation of TGF-β and TLR3, we modulated NF-κB activity through three approaches: plasmid-mediated overexpression, siRNA knockdown, and pharmacological inhibition using BAY 11–7082^41^ (Fig. 5F and G; Fig. S5I and S5J). A concentration gradient (0, 5 μM, 10 μM, 20 μM, 50 μM) of BAY 11–7082 was tested. Toxicity assessment revealed that 20 μM significantly inhibited ECA109 cell proliferation; thus, 10 μM was selected for subsequent experiments (Fig. S5I). Concurrently, treatment with BAY 11–7082 (10 μM) markedly suppressed its nuclear expression (Fig. S5J). Notably, plasmid-mediated NF-κB overexpression promoted the expression of both TGF-β and TLR3, whereas siRNA-mediated NF-κB knockdown or administration of BAY 11–7082 produced the opposite effect, further supporting the regulatory function of NF-κB in this pathway (Fig. 5F and G; Fig. S5K, S5L). To provide direct evidence for NF-κB binding to the promoters of TGF-β and TLR3, we designed the primers targeting the highest-scoring binding sites identified in the database (Table S1). Subsequent ChIP assays confirmed P65 binding at these sites, experimentally supporting the hypothesis that NF-κB serves as a transcriptional regulator for both TLR3 and TGF-β (Fig. 5H and I). In addition, pharmacological blockade of NF-κB activation by BAY 11–7082 led to a significant reduction in its recruitment to the TGF-β and TLR3 promoters, as confirmed by ChIP analysis (Fig. S5M, S5N). Given that HMGB3 and TLR3 can interact and that HMGB3 knockdown inhibits TLR3-induced NF-κB activation, this regulatory mechanism appears to be at least partially dependent on HMGB3 (Fig. 5D and E; Fig. S5D–S5H). Collectively, these findings provide new insights into the molecular cascade linking HMGB3–TLR3 interaction to TGF-β signaling activation in ESCC, highlighting NF-κB as a key transcriptional regulator in this pathway.

To further explore whether HMGB3 regulates the TGF-β or NF-κB pathways through alternative mechanisms, we considered its established role in modulating chromatin conformation. Given that HMGB3 can influence chromatin architecture, we hypothesized that it might exert its regulatory effects by altering chromatin structure. To test this, we conducted an assay for transposase-accessible chromatin with high-throughput sequencing (ATAC-seq) analysis following HMGB3 knockdown. The results revealed that 1605 peaks showed decreased chromatin accessibility, while 2148 peaks exhibited increased accessibility (|fold change| ≥ 2, false discovery rate < 0.05) (Fig. S5O). Notably, genes associated with the TGF-β pathway, including TGF-β receptor 2 (TGFBR2), TGF-β receptor 3 (TGFBR3), and SMAD6, displayed marked changes in chromatin accessibility, whereas no significant alterations were observed for NF-κB-related genes (Table S6). Complementing these findings, KEGG pathway analysis further strengthened the connection between HMGB3 and oncogenic processes by highlighting its involvement in “pathways in cancer” (Fig. S5P). These collective results suggest a model wherein HMGB3 may orchestrate gene expression programs, particularly those related to TGF-β signaling, through direct modulation of chromatin structure. However, the detailed molecular mechanisms governing this regulatory axis warrant further investigation to fully elucidate HMGB3’s role in chromatin-mediated transcriptional control.

### TGF-β is indispensable for promoting *in vitro* cell proliferation, invasion, and migration in ESCC mediated by the TGIF2-HMGB3 axis

**Figure 6:**
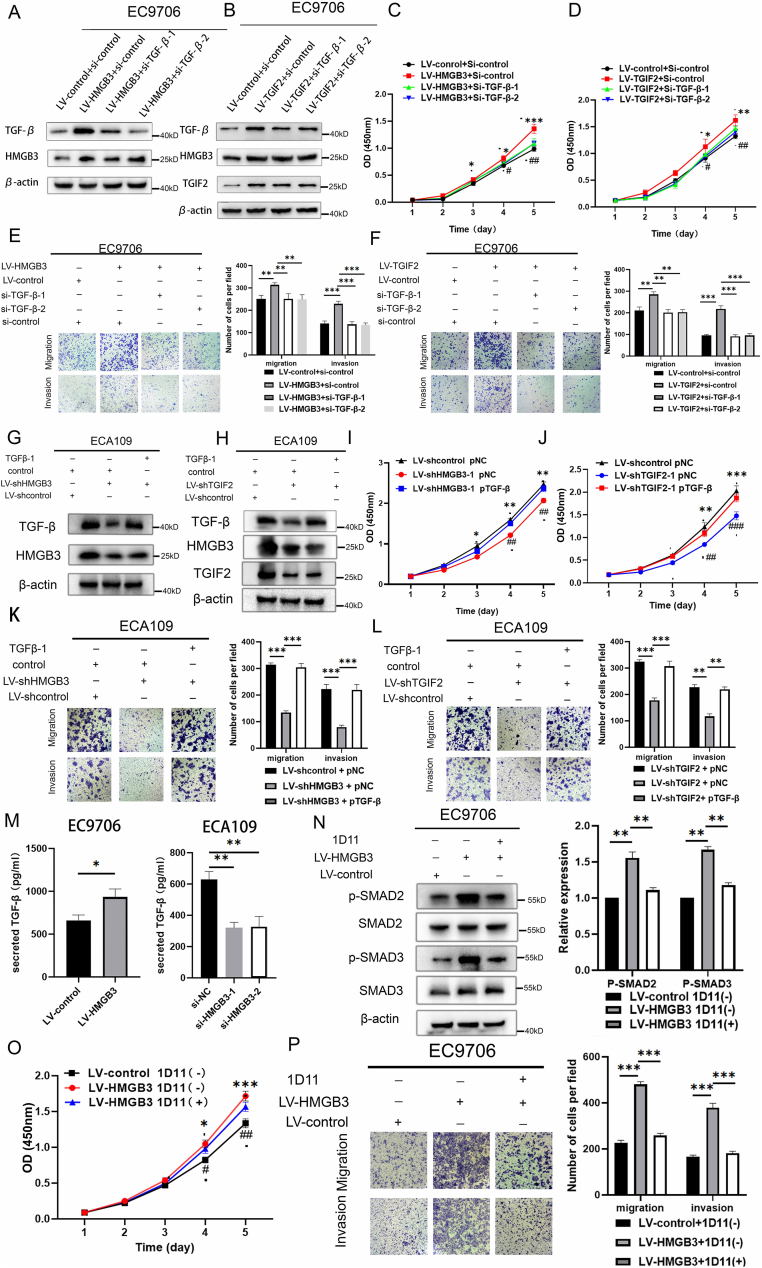
TGF-β is indispensable for the promotion of cell proliferation and metastasis in esophageal squamous cell carcinoma (ESCC) driven by the TGIF2–HMGB3 axis. **(A, B)** Relative protein levels of HMGB3, TGIF2, and TGF-β in EC9706 cells were analyzed via Western blotting after lentivirus infection and siRNA transfection. **(C, D)** The proliferative capability of ESCC cells after modifying the expression of HMGB3/TGF-β or TGIF2/TGF-β was demonstrated by the CCK-8 assay, with optical density (OD) measurements recorded daily over a span of 5 days (“∗” denotes a comparison between LV-control plus si-control and LV-HMGB3/TGIF2 plus si-control, whereas “^#^” signifies a comparison between LV-HMGB3/TGIF2 plus si-control and LV-HMGB3/TGIF2 plus si-TGF-β). **(E, F)** Migratory and invasive capabilities of ESCC cells subsequent to modifying the HMGB3/TGF-β or TGIF2/TGF-β expression were demonstrated by the Transwell analysis (upper panel), with the quantification of migrated cells performed (lower panel). **(G)** Protein levels of HMGB3 and TGF-β in EC9706 cells were analyzed by Western blotting after lentivirus infection and plasmid DNA (pDNA) transfection. **(H)** Protein levels of HMGB3, TGIF2, and TGF-β in EC9706 cells were analyzed by Western blotting following lentivirus infection and plasmid DNA (pDNA) transfection. **(I, J)** The proliferative capacity of ESCC cells following the modulation of HMGB3/TGF-β or TGIF2/TGF-β expression was evaluated by the CCK-8 assay, with OD measurements recorded daily for 5 days (“∗” denotes a comparison between LV-shcontrol plus pNC and LV-shHMGB3/TGIF2 plus pNC, whereas “^#^” signifies a comparison between LV-shHMGB3/TGIF2 plus pNC and LV-shHMGB3/TGIF2 plus pTGF-β). **(K, L)** The Transwell analysis delineates migratory and invasive capabilities of ESCC cells subsequent to modifying the HMGB3/TGF-β or TGIF2/TGF-β expression (left panel), with quantification of migrated and invasive cells performed (right panel). **(M)** Secreted levels of TGF-β in ESCC cells with varying levels of HMGB3 expression were measured using ELISA. **(N)** The block of SMAD-dependent TGF-β signaling by 1D11, a TGF-β neutralizing agent, was assessed using Western blotting. **(O)** The CCK-8 assay was employed to evaluate the proliferative capacity of ESCC cells following the blockade of secreted TGF-β, with OD measurements recorded daily for 5 days (“∗” represents a comparison between LV-control plus 1D11 [−] and LV-HMGB3 plus 1D11 [−], whereas “^#^” denotes a comparison between LV-HMGB3 plus 1D11 [−] and LV-HMGB3 plus 1D11 [−]). **(P)** The Transwell analysis was utilized to assess the migrative and invasive capabilities of ESCC cells following the blockade of secreted TGF-β, with the quantification of migrated cells performed (right panel). All data were expressed as mean ± standard deviation. Statistical significance is denoted as ∗∗∗*P* < 0.001, ∗∗*P* < 0.01, and ∗*P* < 0.05, ^###^*P* < 0.001, ^##^*P* < 0.01, and ^#^*P* < 0.05.

Next, we determined whether the impact of TGF-β on the TGIF2-HMGB3 axis induced proliferation, invasion, and migration of ESCC cells. HMGB3- or TGIF2-overexpressing EC9706 cells were respectively transfected with si-TGF-β-1 or si-TGF-β-2. TGIF2, HMGB3, and TGF-β expression were qualified by WB (Fig. 6A and B). Significantly, the *in vitro* CCK-8 assay (Fig. 6C and D) indicated that reducing TGF-β levels hindered the ability of the TGIF2/HMGB3 axis to drive ESCC cell proliferation. Moreover, the Transwell assay (Fig. 6E and F) demonstrated that the up-regulation of the TGIF2/HMGB3 axis, which initially enhanced the invasion and migration capabilities of ESCC cells, was mitigated via TGF-β down-regulation. We next overexpressed TGF-β using a plasmid in HMGB3-down-regulated or TGIF2-down-regulated ECA109 cells. TGIF2, HMGB3, and TGF-β expression were assessed by WB (Fig. 6G and H). Subsequently, the CCK-8 assay (Fig. 6I and J) and Transwell assay (Fig. 6K and L) demonstrated that ESCC cell proliferation, invasion, and migration capabilities decreased upon the inactivation of the TGIF2/HMGB3 axis; this effect was reversed upon elevating TGF-β levels.

Moreover, the ELISA assay revealed that TGF-β up-regulation, induced by the activation of the TGIF2/HMGB3 axis, improved the secreted TGF-β levels in the culture medium (Fig. 6M). The WB analysis demonstrated that 1D11, a TGF-β-neutralizing antibody, effectively inhibited TGF-β secretion in the medium (Fig. 6N). Furthermore, blocking TGF-β secretion reversed the heightened proliferation (Fig. 6O) and metastasis (Fig. 6P) abilities induced by TGIF2/HMGB3/TLR3 axis activation. The TGIF2/HMGB3/TLR3 axis might partially execute its function by enhancing TGF-β secretion.

#### HMGB3 expression is positively associated with TGIF2 and TGF-β expression in human ESCC specimens

**Figure 7:**
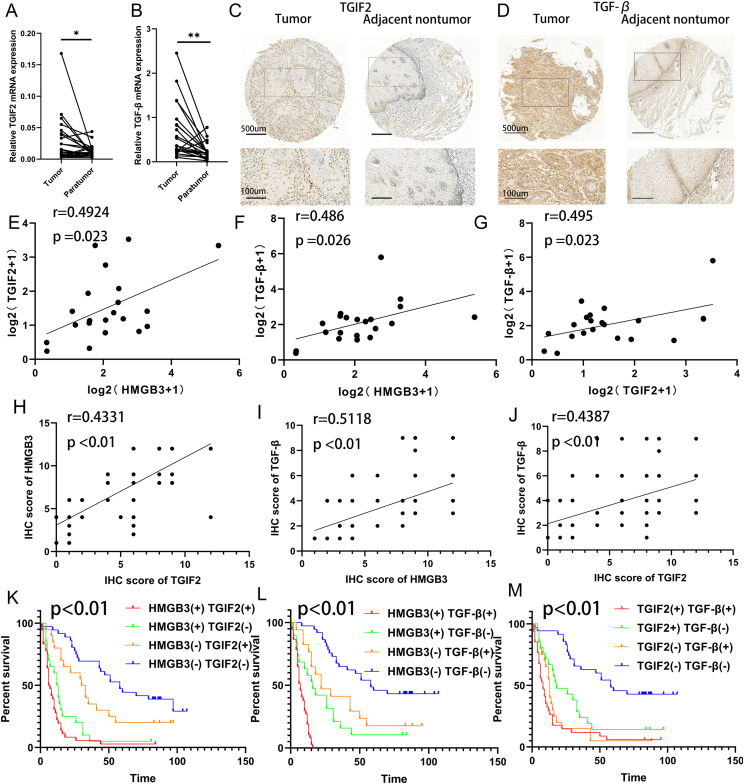
Clinical significance and correlation among HMGB3, TGIF2, and TGF-β. **(A)** Differential TGIF2 expression in 20 pairs of esophageal squamous cell carcinoma (ESCC) and their respective paratumor tissues was revealed by quantitative PCR analysis. **(B)** Quantitative PCR analysis unveils varying expression of TGF-β in 20 pairs of ESCC and their corresponding paratumor tissues. **(C)** Representative immunohistochemistry images depicting the staining of TGIF2 in both ESCC and their corresponding paratumor tissues. **(D)** Representative immunohistochemistry image illustrating TGF-β staining in both ESCC and their corresponding paratumor tissues. **(E)** Correlation analysis between the relative mRNA expression of HMGB3 and TGIF2 in ESCC tissues (*n* = 20). **(F)** Analysis of the association between the relative mRNA expression of TGF-β and HMGB3 in ESCC tissues (*n* = 20). **(G)** Correlation between the relative mRNA expression of TGIF2 and TGF-β in ESCC tissues (*n* = 20). **(H)** Correlation between the relative mRNA expression of TGIF2 and HMGB3 in ESCC tissues (*n* = 112). **(I)** Correlation between the relative mRNA expression of HMGB3 and TGF-β in ESCC tissues (*n* = 112). **(J)** Correlation between the relative mRNA expression of TGIF2 and TGF-β in ESCC tissues (*n* = 112). **(K)** Kaplan–Meier analysis reveals how the combined expression of HMGB3 and TGIF2 proteins impacts overall survival in patients with ESCC (*n* = 112). The red line represents patients with higher expression of TGIF2 and HMGB3, whereas the blue line represents those with lower expression of TGIF2 and HMGB3. The green line depicts patients with lower TGIF2 and higher HMGB3 expression. Additionally, the yellow line represents those with higher TGIF2 and lower HMGB3 expression. **(L)** Kaplan–Meier analysis reveals how the combined expression of HMGB3 and TGF-β proteins impacts overall survival in patients with ESCC (*n* = 112). The red line represents patients with higher HMGB3 and TGF-β expression, whereas the blue line indicates those with lower HMGB3 and TGF-β expression. Additionally, the green line indicates patients with higher HMGB3 and lower TGF-β expression, and the yellow line represents patients with lower HMGB3 and higher TGF-β expression. **(M)** Kaplan–Meier analysis reveals how the combined expression of HMGB3 and TGF-β proteins impacts overall survival in patients with ESCC (*n* = 112). The red line indicates the group with higher TGIF2 and TGF-β expression, whereas the blue line represents patients with lower TGIF2 and TGF-β expression. Additionally, the green line indicates patients with higher TGIF2 and lower TGF-β expression, and the yellow line represents patients with lower expression of TGIF2 and higher expression of TGF-β. All data were expressed as mean ± standard deviation. Statistical significance is denoted as ∗∗∗*P* < 0.001, ∗∗*P* < 0.01 and ∗*P* < 0.05.

**Figure S6:**
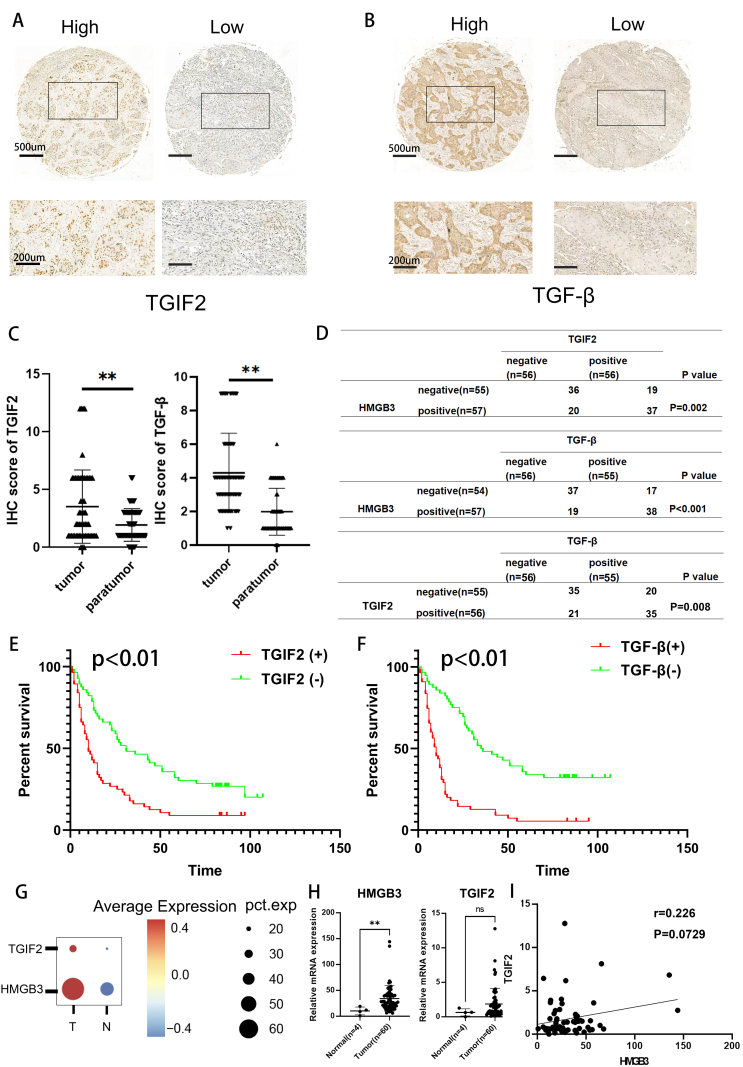
Clinical significance and correlation between TGIF2, HMGB3, and TGF-β. **(A)** Representative immunohistochemistry (IHC) images illustrating high or low levels of TGIF2 expression in esophageal squamous cell carcinoma (ESCC) tissues. **(B)** Representative IHC images illustrating high or low levels of TGF-β expression in ESCC tissues. **(C)** Semiquantitative analysis was utilized to assess the IHC scores of TGIF2 in ESCC and their matched paratumor tissues (*n* = 57 pairs). Semiquantitative analysis was utilized to evaluate the IHC scores of TGF-β in ESCC and their matched paratumor tissues (*n* = 57 pairs). **(D)** Correlation analysis of TGIF2/HMGB3, TGF-β/HMGB3, or TGIF2/TGF-β in ESCC tissues (*n* = 112). **(E)** The correlation between mRNA expression levels of TGIF2 and overall survival in ESCC patients was demonstrated by the Kaplan–Meier analysis (*n* = 112). Patients with high TGIF2 expression are represented by the red line, whereas those with low TGIF2 expression are represented by the green line. **(F)** Kaplan–Meier analysis demonstrates the correlation between expression levels of TGF-β and overall survival in ESCC patients (*n* = 112). Patients with high TGF-β expression are represented by the red line, whereas those with low TGF-β expression are represented by the green line. **(G)** Dot plots showing the expression of HMGB3 and TGIF2 in tumor epithelial cells versus normal epithelial cells in GSE160269. Dot size represents the proportion of cells expressing the gene (larger = higher percentage). Color intensity (red scale) represents the average expression level in expressing cells (darker red = higher expression). **(H)** Bar graph comparing the expression of HMGB3 and TGIF2 between normal epithelial cells and tumor epithelial cells in GSE160269. Each point represents the average expression in epithelial cells from one sample. **(I)** Spearman correlation analysis of TGIF2 expression and HMGB3 expression in epithelial cells (*n* = 64) in GSE160269. Each point represents the average expression in epithelial cells from one sample. Statistical significance is denoted as ∗∗∗*P* < 0.001, ∗∗*P* < 0.01, and ∗*P* < 0.05.

To elucidate the clinical significance of TGIF2 and TGF-β, we analyzed their expression profiles in ESCC cohorts. We assessed the mRNA levels of TGIF2 and TGF-β in 20 matched adjacent non-tumor and primary ESCC specimens (Fig. 7A and B). TGIF2 (*P* < 0.05) and TGF-β (*P* < 0.01) expression were higher in primary ESCC than in adjacent non-tumor tissues. The IHC results showed trends similar to those of qRT-PCR results (Fig. 7C and D; Fig. S6A–S6C). The correlation analysis of qRT-PCR (Fig. 7E–G) and IHC (Fig. 7H–J) revealed a positive association between TGIF2, HMGB3, and TGF-β. The chi-squared test revealed an intricate correlation between HMGB3 overexpression and elevated TGIF2 or TGF-β expression (Fig. S6D). Similarly, a higher TGF-β expression was associated with TGIF2 overexpression. According to IHC analysis, we found that TGIF2 overexpression was strongly associated with a higher pathological grade (*P* = 0.006). TGF-β overexpression was intricately related to higher pathological grade (*P* = 0.037). Concurrently, higher TGF-β expression was observed in the male group (*P* = 0.042) (Table 1). Additionally, patients demonstrating positive TGIF2 or TGF-β expression had reduced OS compared with those with negative TGIF2 or TGF-β expression (Fig. S6E and S6F). Those exhibiting dual positive expression of TGIF2/HMGB3, HMGB3/TGF-β, or TGIF2/TGF-β had the poorest outcomes (Fig. 7K–M).

Using IHC data, we assessed the prognostic significance of TGIF2, HMGB3, and TGF-β in ESCC via univariate and multivariate Cox regression analyses (Table 2). The univariate analysis revealed that the AJCC stage (*P* < 0.001), tumor invasion (*P* = 0.001), age (*P* = 0.003), lymph node metastasis (*P* = 0.006), TGIF2 expression (*P* < 0.001), HMGB3 expression (*P* < 0.001), and TGF-β expression (*P* < 0.001) were significant prognostic factors for OS in ESCC. The multivariate analysis demonstrated that sex (*P* = 0.007), tumor invasion (*P* = 0.034), HMGB3 expression (*P* < 0.001), TGF-β expression (*P* < 0.001), and TGIF2 expression (*P* = 0.046) were independent prognostic risk factors for OS in patients with ESCC.

**Table 2:** Univariate and multivariate analysis of the factors associated with overall survival in esophageal squamous cell carcinoma patients.

Variables (*n* = 112)	Overall survival					
	Univariate analysis			Multivariate analysis		
	Hazard ratio	95% confidence interval	*P* value	Hazard ratio	95 % confidence interval	*P* value
Age (≤65 versus >65)	2.177	1.295–3.658	**0.003**	1.594	0.948–2.682	0.079
Sex (male versus female)	1.011	0.671–1.524	0.958	2.511	1.282–4.917	**0.007**
Pathological grade (I/II versus III)	0.743	0.444–1.243	0.258	1.025	0.586–1.794	0.931
Tumor size (≤5 versus >5)	1.55	0.988–2.432	0.057	1.208	0.729–2.003	0.464
Tumor invasion (T1–T2 versus T3–T4)	2.661	1.491–4.750	**0.001**	2.255	1.062–4.790	**0.034**
Lymph node metastasis (absent versus present)	1.8	1.184–2.737	**0.006**	0.926	0.277–3.094	0.901
American joint committee on cancer stage (I–II versus III–IV)	3.163	2.004–4.991	**<0.001**	1.544	0.410–5.819	0.521
HMGB3 expression (low versus high)	4.914	3.135–7.705	**<0.001**	3.785	2.071–6.917	**<0.001**
TGIF2 expression (low versus high)	2.325	1.525–3.543	**<0.001**	1.680	1.009–2.797	**0.046**
TGF-β expression (low versus high)	3.448	2.228–5.535	**<0.001**	3.100	1.841–5.220	**<0.001**

To further elucidate the clinical relevance of TGIF2 and HMGB3 in ESCC, we analyzed a publicly available single-cell RNA sequencing dataset (GSE160269), which includes 60 ESCC samples and 4 normal esophageal tissues.^42^ Dot plot analysis revealed that both HMGB3 and TGIF2 were highly expressed in epithelial cells from ESCC compared with normal tissues, with a higher proportion of positive cells in tumor samples (Fig. S6G). Furthermore, after averaging the expression levels of HMGB3 and TGIF2 across all epithelial cells in each sample, we observed significantly elevated expression of HMGB3 (*P* < 0.01) in ESCC versus normal tissues (Fig. S6H). Additionally, correlation analysis was also conducted between HMGB3 and TGIF2 expression (*r* = 0.226, *P* = 0.0729), further supporting their possible functional interplay in ESCC pathogenesis (Fig. S6I).

## Discussion

Recent years have witnessed significant improvements in ESCC diagnosis and treatment.^43^ However, the prognosis of patients with ESCC often falls short of expectations, and uncontrolled proliferation and metastasis remain significant challenges in its management. Therefore, it is crucial to investigate the mechanisms underlying ESCC proliferation and metastasis. Recent studies have increasingly highlighted HMGB3 as a key tumor regulator^12^^,^^14^ and have indicated its potential significance in understanding and targeting these processes in ESCC. In laryngeal squamous cell carcinoma, HMGB3 induces cell proliferation and metastasis by targeting E2F transcription factor 1 (E2F1).^44^ The presence of nuclear exosomal HMGB3 in nasopharyngeal carcinoma cells correlates with metastasis induction by promoting angiogenesis.^45^ Additionally, HMGB3 was reported to promote glycolysis by activating the β-catenin pathway in nasopharyngeal carcinoma cells.^46^ Furthermore, HMGB3 promotes resistance to poly(ADP-ribose) polymerase (PARP) inhibitors (PARPi) through its interaction with PARP1, and targeted inhibition of HMGB3 may represent a strategy to overcome PARPi resistance in ovarian cancer therapy.^47^ We confirmed that HMGB3 is up-regulated in ESCC. Additionally, its high expression was positively associated with enhanced proliferation and metastasis. The mRNA and protein levels of HMGB3 were higher in ESCC tissues than in non-tumor tissues. Thus, HMGB3, as a newly identified marker with therapeutic potential, holds promise for predicting malignant progression and guiding treatment strategies for ESCC.

We investigated the upstream and downstream mechanisms of HMGB3 in ESCC development and progression. TGIF2 was identified as an upstream transcription factor of HMGB3, enhancing its expression and augmenting its oncogenic potential. TGIF2 promotes the malignant progression of breast cancer^21^^,^^22^; it functions as a transcription factor regulating key genes, such as FUT8^24^ and OCT4.^23^ TGIF2 has been implicated in the proliferation and metastasis of lung adenocarcinoma,^23^ melanoma,^24^ and colorectal cancer.^25^ Furthermore, the EGFR signaling-mediated phosphorylation of TGIF2 enhances its function as a transcription factor, thereby accelerating lung adenocarcinoma progression.^23^ It is a critical regulator of proliferation, invasion, and metastasis across a spectrum of tumors, highlighting its pivotal function in the progression of different cancers.^26^^,^^27^ However, its contribution to ESCC progression remains unclear. We discovered a significant association between elevated levels of TGIF2 and higher pathological grades, as well as increased proliferation and metastatic capabilities in ESCC. Furthermore, TGIF2-driven progression of ESCC was mitigated by HMGB3 down-regulation. Conversely, the ectopic overexpression of HMGB3 counteracted the inhibitory effects on ESCC cell proliferation and metastasis resulting from TGIF2 deletion. Additionally, a positive association between TGIF2 and HMGB3 expression was noted, suggesting that TGIF2 partially promotes ESCC proliferation and metastasis by up-regulating HMGB3 expression. Thus, TGIF2 not only plays a crucial role in ESCC development but also is a prognostic marker for the disease.

HMGB3 is involved in several signaling pathways, including the mTOR,^48^ Wnt,^20^ and DNA damage signaling pathways.^12^ However, reports on the downstream targets and associated pathways are scarce.^18^ We used RNA sequencing to comprehensively analyze the potential downstream targets or pathways influenced by HMGB3. The KEGG enrichment analysis results underscored a potential mechanism that HMGB3 might activate the TGF-β signaling pathway. TGF-β signaling operates via both Smad-dependent and Smad-independent mechanisms, embodying a dual function during tumorigenesis.^49^^,^^50^ TGF-β signaling can suppress the malignant progression of normal cells,^51^ whereas it functions as an oncogene during tumor progression.^52^ This “double-edged sword” phenomenon may be attributed to the evasion of immune detection,^51^ mobilization of myofibroblasts,^53^ and mobilization of osteoclasts.^53^ Recent research has uncovered that TGF-β signaling impacts the development of ESCC.^54^^,^^55^ For instance, TGF-β/Smad2 signaling facilitates the malignant progression of ESCC by promoting the epithelial–mesenchymal transition.^54^ Additionally, the TGF-β signaling pathway can stimulate the growth and dissemination of ESCC by establishing a positive feedback loop involving zinc finger E-box binding homeobox 1 (ZEB1).^55^ Therefore, targeting the TGF-β signaling pathway could be a promising strategy to curb tumor proliferation and metastasis in ESCC. We identified that both TGIF2 and HMGB3 positively activated Smad-dependent TGF-β signaling within ESCC. TGIF2-induced activation of TGF-β signaling and its oncogenic effects are significantly dependent on HMGB3. Recovery experiments confirmed the pivotal function of the TGIF2/HMGB3/TGF-β axis in promoting growth and metastasis. Our results unveil a novel mechanistic insight into how the TGIF2/HMGB3/TGF-β signaling pathway orchestrates the regulation of proliferation, invasion, and metastasis, thereby accelerating ESCC progression.

To comprehensively understand how HMGB3 influences TGF-β signaling in ESCC, we used the differentially expressed genes identified from the RNA sequencing analysis. One significantly down-regulated gene was TLR3, which prompted our interest in this topic. The TLR family members have been implicated in HMGB1-induced inflammation and carcinoma,^29^^,^^30^ which possesses a biochemical structure similar to that of HMGB3.^7^ TLR3 is intricately linked to the cancer-promoting impact of HMGB3. Furthermore, research indicates that TLR3 positively regulates TGF-β signaling in different cancers, such as neuroblastoma^31^ and breast cancer.^32^ Therefore, we speculated that TLR3 could facilitate TGF-β signaling in ESCC, potentially serving as a link between HMGB3 and the TGF-β pathway. The subsequent studies validated our hypotheses. Our data demonstrated that poly (I:C)-induced activation of TLR3 could amplify the expression of crucial molecules in a Smad-dependent TGF-β signaling pathway, suggesting that TLR3 can activate TGF-β signaling in ESCC cells. Furthermore, our research revealed that HMGB3 and TGIF2 could stimulate the expression of TLR3, with TGIF2’s regulatory function in TLR3 expression being partially dependent on HMGB3. The Co-IP assay revealed a direct interaction between HMGB3 and TLR3. In summary, these findings unveiled a novel TGIF2/HMGB3/TLR3/TGF-β signaling axis in ESCC progression, holding potential clinical significance.

We further explored the mechanisms by which the interaction between HMGB3 and TLR3 regulates the TGF-β signaling pathway. According to the literature on the regulation of carcinogenesis by the interaction of HMGB and TLR families, NF-κB activation is the most consistently reported downstream event following HMGB/TLR family interactions.^39^ As a key transcription factor, NF-κB regulates the expression of several molecules, including TGF-β.^40^^,^^56^ We experimentally confirmed that the interaction between HMGB3 and TLR3 can activate the NF-κB signaling pathway in ESCC cells, and NF-κB may transcriptionally activate the expression of TGF-β and TLR3. This finding may explain the activation of the TGF-β signaling pathway and the transcriptional regulation of TLR3 following the HMGB3 and TLR3 interaction.

We further studied the clinical significance of the TGIF2/HMGB3/TGF-β axis in ESCC, revealing a stark correlation between the high expression of this axis and poor prognosis in ESCC patients. Moreover, we identified a significant association between this pathway and several key clinical features: HMGB3 was significantly correlated with sex, tumor invasion, and AJCC staging; elevated levels of TGIF2 could indicate a higher pathological grade. Similarly, TGF-β expression was linked to sex and pathological grade. Notably, sex, tumor invasion, HMGB3, TGIF2, and TGF-β were identified as independent risk factors for poorer survival outcomes. This study is the first to articulate the pivotal role of the TGIF2/HMGB3/TGF-β axis in predicting ESCC prognosis, suggesting that this pathway may be a promising target for ESCC therapy. However, this hypothesis requires further validation using broader cohort and subgroup analyses.

**Figure 8:**
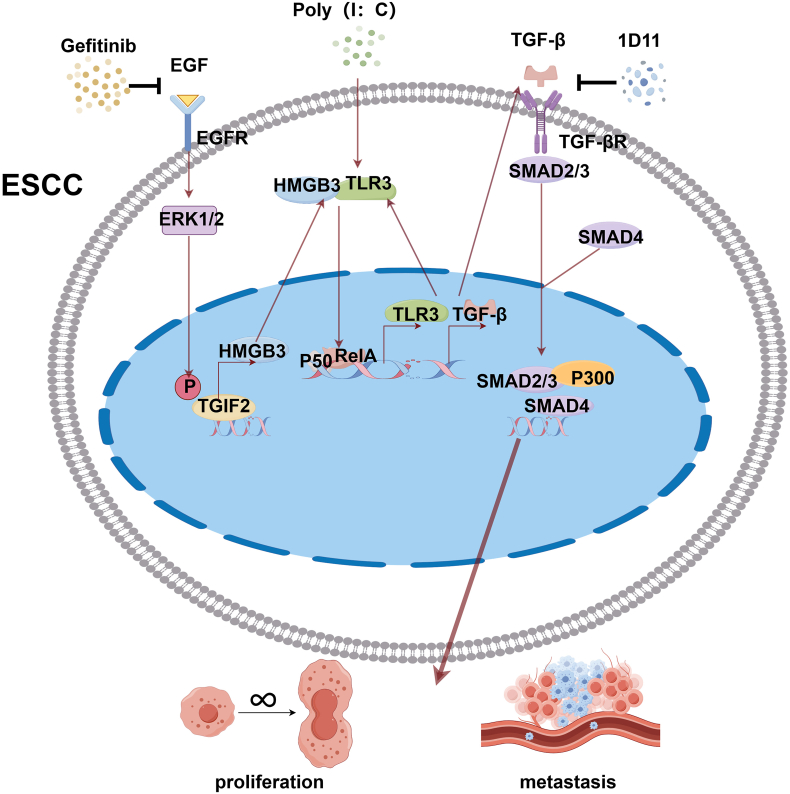
TGIF2-mediated HMGB3 overexpression promotes esophageal squamous cell carcinoma proliferation and metastasis through TLR3/TGF-β signaling.

Collectively, our study provides several insights into the molecular dynamics of ESCC. Both HMGB3 and TGIF2 exhibited elevated expressions in ESCC tissues, correlating with a poor prognosis for affected individuals. Furthermore, our research identified TGIF2 as a key transcription factor for HMGB3, playing a pivotal role in ESCC progression. We observed that heightened levels of either TGIF2 or HMGB3 can trigger the Smad-dependent TGF-β signaling, thereby hastening ESCC development and progression. Notably, the oncogenic TGIF2-facilitated activation of TGF-β signaling appears to be, at least partially, contingent upon the presence of HMGB3. To understand the intricate mechanisms at play, we have verified that TLR3 co-interacts with HMGB3. This interaction is believed to further stimulate TGF-β signaling, underscoring the complexity and interconnectivity of the molecular pathways involved in ESCC. These critical pieces of evidence suggest that a novel TGIF2/HMGB3/TLR3/TGF-β signaling pathway exists (Fig. 8). This discovery not only paves the way for the development of targeted therapeutic strategies but also holds promise for the identification of novel ESCC biomarkers, potentially revolutionizing the approach to diagnosis and treatment in this challenging disease landscape.

Our study had certain limitations. First, although our study has identified HMGB3 as a regulator of TGF-β and the TGF-β pathway through its regulation of and interaction with TLR3, several intriguing questions remain unresolved. We observed that while HMGB3 modulated the protein levels of both SMAD2/3 and their phosphorylated forms, the phosphorylation ratios (p-SMAD2/SMAD2 and p-SMAD3/SMAD3) remained unchanged. Furthermore, RNA sequencing analysis did not detect corresponding transcriptional changes in SMAD2/3 expression, suggesting the existence of complex regulatory mechanisms. The ATAC-seq findings revealing HMGB3-mediated chromatin accessibility changes at key TGF-β pathway components further highlight the multifaceted nature of this regulation. These results collectively suggest that HMGB3 may orchestrate TGF-β signaling through multiple parallel mechanisms, including both canonical pathway activation and chromatin-based regulation. Future investigations should focus on elucidating the molecular details of HMGB3’s chromatin remodeling functions and their specific contributions to TGF-β pathway modulation, which may provide a more comprehensive understanding of this complex regulatory network and HMGB3’s oncogenic function and mechanism. Second, although evidence suggests that HMGB3 can transcriptionally regulate TLR3 and subsequently bind to it, the predominant mechanism driving ESCC development through this interaction remains undetermined. Clarifying whether transcriptional regulation or protein–protein interactions will be vital for unraveling the complex pathology of ESCC. Third, our findings indicate that the TGIF2/HMGB3 axis can enhance the expression and secretion of TGF-β in ESCC, promoting the proliferation and metastasis. However, the dynamics of how ESCC cells interact with the tumor microenvironment following the activation of the TGIF2/HMGB3/TGF-β axis are not fully understood. Investigating the role of TGF-β secreted by different cells within the tumor microenvironment on ESCC cells could provide deeper insights into the disease process and potential therapeutic targets. Moreover, the clinical relevance of the TGIF2/HMGB3/TGF-β axis in ESCC needs to be validated in a larger cohort. Given its correlation with several clinical features, subgroup analyses should be conducted, and a prognosis prediction model incorporating these factors that could offer a more nuanced understanding of personalized treatment strategies must be developed. Besides, while current single-cell sequencing technologies have provided crucial insights for investigating tumor mechanisms and therapeutic strategies, the available single-cell sequencing datasets for ESCC remain limited. Future research should focus on expanding these datasets and conducting comprehensive bioinformatics analyses to further elucidate the functional role of the TGIF2/HMGB3/TGF-β axis in ESCC pathogenesis.

## CRediT authorship contribution statement

**Liaoran Niu:** Writing – review & editing, Writing – original draft, Visualization, Validation, Methodology, Investigation, Formal analysis, Data curation. **Wanli Yang:** Writing – review & editing, Writing – original draft, Project administration, Funding acquisition, Data curation, Conceptualization. **Wei Zhou:** Writing – review & editing, Writing – original draft, Investigation, Data curation, Conceptualization. **Lili Duan:** Visualization, Investigation, Data curation. **Qi Wang:** Investigation, Formal analysis, Data curation. **Xiaoqian Wang:** Visualization, Investigation, Formal analysis. **Yiding Li:** Writing – review & editing, Investigation, Data curation. **Chengchao Xu:** Visualization, Investigation. **Yujie Zhang:** Formal analysis, Data curation. **Jinqiang Liu:** Visualization, Data curation. **Jian Zhang:** Funding acquisition, Methodology, Resources. **Daiming Fan:** Supervision, Resources, Funding acquisition, Conceptualization. **Jianyong Zheng:** Supervision, Resources, Project administration, Methodology, Funding acquisition, Conceptualization. **Liu Hong:** Writing – review & editing, Supervision, Resources, Project administration, Methodology, Funding acquisition, Conceptualization.

## Ethics declaration

The animal experiments were authorized by the Ethics Committee of the Animal Center (the Fourth Military Medical University). Clinical sample experiments received authorization from the Ethics Committee (Xijing Hospital, Fourth Military Medical University), with authorization number KY20212195-F-1.

## Data availability

The Material and Data supporting this manuscript are available from the corresponding author upon reasonable request. All requests will be subject to restrictions related to human biobank protection and GDPR legislation on personal data.

## Funding

This study was partially funded by grants from the 10.13039/501100001809National Natural Science Foundation of China (No. 82073210, 82372693, 82303427), the National Clinical Research Center for Digestive Diseases (China) (No. 2015BAI13B07), the Shaanxi Provincial Outstanding Youth Fund (China) (No. 2024JC-JCQN-77), and the Fund for Scientific and Technological Innovation Team of Shaanxi Innovation Capability Support Plan (China) (No. 2023-CX-TD-67).

## Conflict of interests

The authors declared no competing interests.

## Figures and Tables

**figs1:**
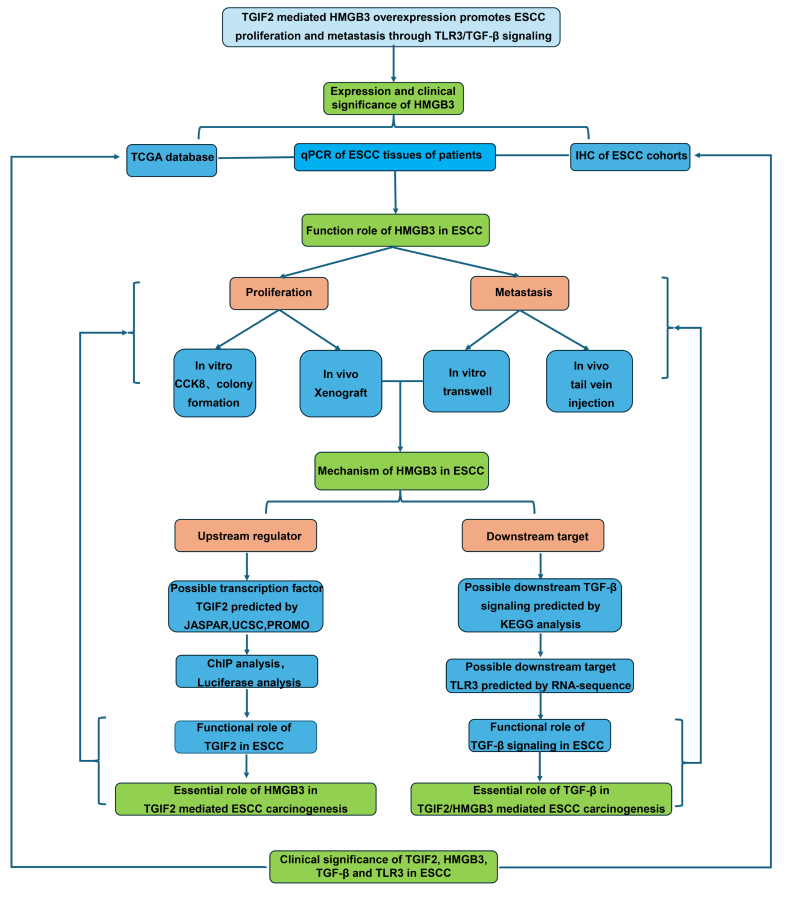
**Figure S1** The flow chart for study design.

**figs2:**
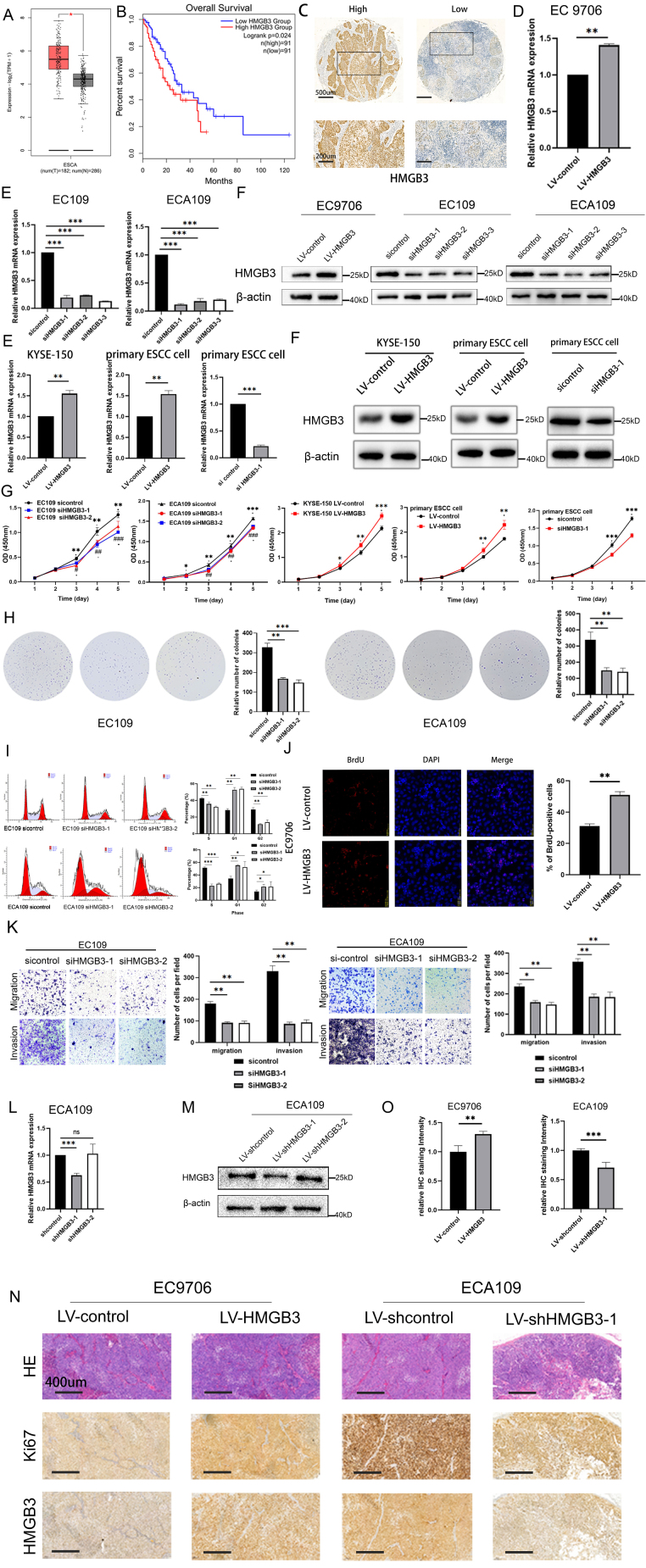
**Figure S2** Overexpression of HMGB3 promotes esophageal squamous cell carcinoma (ESCC) proliferation and metastasis and indicates a poor prognosis. **(A)** TCGA datasets indicate the HMGB3 mRNA expression levels in ESCA patients (*n* = 182) versus normal individuals (*n* = 286). **(B)** The Kaplan-Meier analysis reveals the correlation between the mRNA expression of HMGB3 and overall survival in ESCA from the TCGA database. The blue line depicts individuals with lower expression of HMGB3, whereas the red line represents those with higher expression of HMGB3. **(C)** Representative immunohistochemistry images depicting high and low HMGB3 expression in ESCC tissues. **(D)** The quantitative PCR analysis was employed to examine the mRNA expression level of HMGB3 in EC9706 cells following lentivirus infection. **(E)** The quantitative PCR analysis was conducted to assess the expression level of HMGB3 mRNA in EC109, ECA109, KYSE-150 and primary ESCC cell after si-RNA transfection or lentivirus infection. **(F)** Western blotting was conducted to assess the relative protein expression of HMGB3 in EC109, ECA109, EC9706, KYSE-150, and primary ESCC cells, following either infection with lentivirus or siRNA transfection. **(G)** The CCK-8 assay reveals changes in the proliferative capability of ESCC cells following the down-regulation of HMGB3, with the OD measured daily over 5 days. **(H)** The colony formation assay shows how the proliferative capability of ESCC cells changes following the down-regulation of HMGB3, by counting and comparing the colony number in each experimental group. **(I)** Flow cytometry analysis examines the impact of HMGB3 down-regulation on the cell cycle (left), with the percentages of cells in the G1, G2, and S phases determined and compared (right). **(J)** The BrdU incorporation assay demonstrates the effect of HMGB3 overexpression on the S phase of the cell cycle (left), with the relative BrdU incorporation levels being quantified and compared accordingly (right). **(K)** Transwell analysis demonstrates the migratory and invasive capacities of ESCC cells after down-regulation of HMGB3 (left), while quantifying and comparing the migrated and invasive cell numbers (right). **(L)** The quantitative PCR analysis was utilized to examine the relative mRNA expression level of HMGB3 in ECA109 cells following lentivirus infection. **(M)** The relative HMGB3 protein expression in ECA109 cells after lentivirus infection was analyzed using Western blotting. **(N)** Representative images depicting hematoxylin-eosin staining and immunohistochemistry staining for both Ki67 and HMGB3 in xenograft mouse tumor tissues. **(O)** Ki67 expression levels were quantified, and the relative Ki67 levels were calculated. All data were expressed as mean ± standard deviation. Statistical significance is denoted as ∗∗∗*P* < 0.001, ∗∗*P* < 0.01, and ∗*P* < 0.05.

**Figure:**
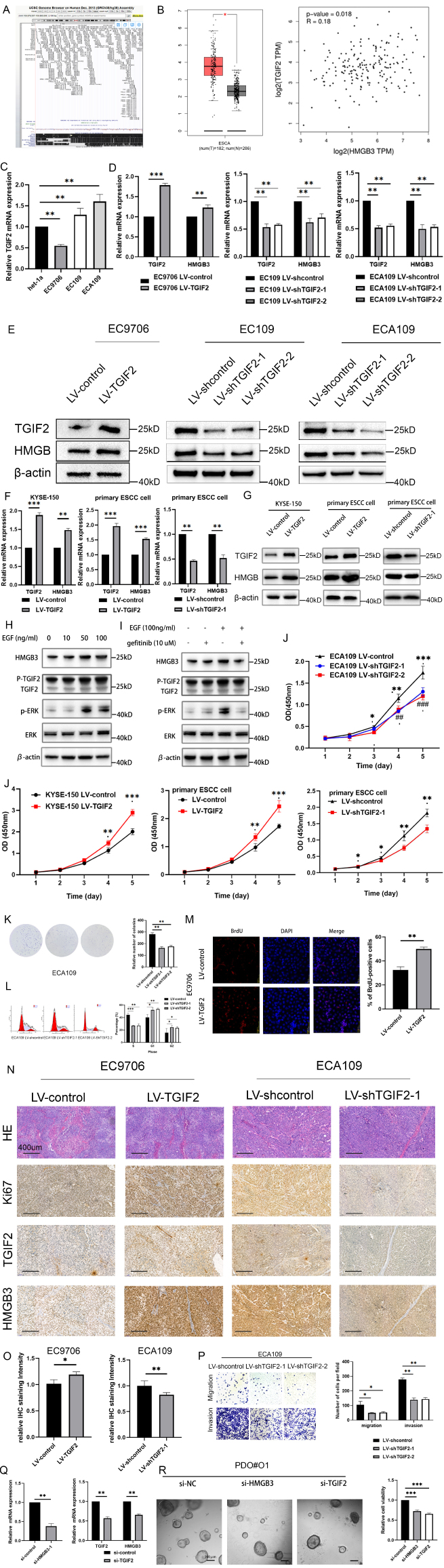
figs3**Figure S3** TGIF2 transcriptionally regulates HMGB3 by binding to the promoter and promotes esophageal squamous cell carcinoma (ESCC) proliferation and metastasis. **(A)** JAPAR and UCSC databases were employed to predict the transcriptional characteristics of HMGB3. **(B)** TCGA datasets indicate the TGIF2 mRNA expression levels in ESCA patients (*n* = 182) versus normal individuals (*n* = 286). Correlation analysis between TGIF2 and HMGB3 expression levels within the TCGA database was also analyzed. **(C)** Identification of the mRNA expression levels of TGIF2 in ESCC cell lines versus het-1A employing quantitative PCR analysis. **(D)** The relative mRNA expression of HMGB3 and TGIF2 in EC109 and EC9706 after infection with lentivirus was analyzed via quantitative PCR. **(E)** The alteration of protein expression levels of TGIF2 and HMGB3 in EC109, ECA109, and EC9706 after lentivirus infection was assessed via Western blotting. **(F)** The mRNA expression levels of HMGB3 and TGIF2 in KYSE-150 and a primary ESCC cell following lentiviral infection were quantified using quantitative PCR. **(G)** Western blotting analysis was performed to evaluate changes in TGIF2 and HMGB3 protein expression in KYSE-150 and a primary ESCC cell after lentiviral infection. **(H)** The protein expression levels of p-TGIF2, HMGB3, ERK, and p-ERK in EC9706 cells were evaluated by Western blotting following treatment with varying concentrations of EGF (0, 10, 50, and 100 ng/mL). **(I)** The protein expression levels of p-TGIF2, HMGB3, ERK, and p-ERK in EC9706 cells were evaluated by Western blotting following co-treatment with EGF (100 ng/mL) and gefitinib (10 μM). **(J)** The CCK-8 assay demonstrates the proliferative capability of ESCC cells following down-regulation of TGIF2 expression, with optical density (OD) measurements recorded daily for 5 days. **(K)** The proliferative ability of ESCC cells was evaluated after down-regulation of TGIF2 expression through colony formation assays (left), comparing colony counts across specified groups (right). **(L)** Flow cytometry analysis was conducted to assess the impact of TGIF2 down-regulation on the cell cycle distribution (left). The percentage of cells in the G1, G2, and S phases was calculated and analyzed accordingly (right). **(M)** The BrdU incorporation assay demonstrates the effect of TGIF2 overexpression on the S phase of the cell cycle (left), with the relative BrdU incorporation levels being quantified and compared accordingly (right). **(N)** Images displaying hematoxylin-eosin staining and immunohistochemistry staining for Ki67, HMGB3, and TGIF2 in xenograft mouse tumor tissues. **(O)** Ki67 expression levels were quantified, and the relative Ki67 levels were calculated. **(P)** The Transwell analysis demonstrates the migratory and invasive potential of ESCC cells after TGIF2 expression down-regulation (top), with counts and comparisons of migrated and invasive cells (bottom). **(Q)** The mRNA expression levels of HMGB3 and TGIF2 in ESCC organoids following siRNA transfection were evaluated using quantitative PCR. **(R)** Representative images of ESCC organoids after treatment with si-control, si-HMGB3, or si-TGIF2. Scale bars: 200 μm. **(S)** ESCC organoids were treated with si-control, si-HMGB3, or si-TGIF2. Cell viability in ESCC organoids was assessed using the CTG-3D assay (*n* = 5). All data were expressed as mean ± standard deviation. Statistical significance is denoted as ∗∗∗*P* < 0.001, ∗∗*P* < 0.01, and ∗*P* < 0.05.
