# Supplementary material for: TGIF2-mediated HMGB3 overexpression promotes esophageal squamous cell carcinoma proliferation and metastasis through TLR3/TGF-β signaling
Source: Genes Dis. 2025 Dec 15;13(3):101987. doi: 10.1016/j.gendis.2025.101987 (PMC12914543; doi:10.1016/j.gendis.2025.101987)
Supplement: Multimedia component 1 [file mmc1.docx]

Table S1 The clinical characteristics of 20 paired paraffin-embedded pathological specimens of ESCC

| **Sex** | **Age** | **Pathological grade** | **T** | **N** | **M** | **AJCC** |
| --- | --- | --- | --- | --- | --- | --- |
| Male | 72 | Ⅰ-Ⅱ | T3 | N0 | M0 | 2B |
| Male | 68 | Ⅰ-Ⅱ | T2 | N0 | M0 | 2B |
| Male | 71 | Ⅰ-Ⅱ | T2 | N0 | M0 | 2B |
| Male | 68 | Ⅱ | T3 | N0 | M0 | 2B |
| Male | 53 | Ⅱ | T2 | N0 | M0 | 2B |
| Female | 64 | Ⅱ | T3 | N0 | M0 | 2B |
| Female | 73 | Ⅰ-Ⅱ | T3 | N0 | M0 | 2A |
| Male | 68 | Ⅰ-Ⅱ | T2 | N0 | M0 | 2B |
| Female | 55 | Ⅱ | T3 | N0 | M0 | 2A |
| Male | 79 | Ⅰ-Ⅱ | T3 | N0 | M0 | 2B |
| Male | 57 | Ⅰ-Ⅱ | T2 | N1 | M0 | 2B |
| Female | 71 | Ⅱ | T3 | N0 | M0 | 2A |
| Female | 68 | Ⅰ-Ⅱ | T3 | N0 | M0 | 2B |
| Female | 63 | Ⅱ | T3 | N0 | M0 | 2B |
| Male | 76 | Ⅰ-Ⅱ | T2 | N1 | M0 | 2B |
| Male | 76 | Ⅱ | T1 | N0 | M0 | 1 |
| Male | 69 | Ⅱ | T2 | N1 | M0 | 2B |
| Male | 51 | Ⅱ | T2 | N0 | M0 | 2B |
| Female | 62 | Ⅱ | T2 | N0 | M0 | 2B |
| Female | 57 | Ⅱ | T2 | N0 | M0 | 2B |
