# Supplementary material for: TGIF2-mediated HMGB3 overexpression promotes esophageal squamous cell carcinoma proliferation and metastasis through TLR3/TGF-β signaling
Source: Genes Dis. 2025 Dec 15;13(3):101987. doi: 10.1016/j.gendis.2025.101987 (PMC12914543; doi:10.1016/j.gendis.2025.101987)
Supplement: Multimedia component 2 [file mmc2.docx]

| GENE | forward | reverse |
| --- | --- | --- |
| HMGB3 | 5′-CCAAAGGGCAAGATGTCCG-3′ | 5′-TTGACAGGGACCTCTGGGTTT-3′ |
| TGIF2 | 5′-TACTTGCACCGCTACAACGC-3′ | 5′- TCCTTCCGAAGCATGTCTGG -3′ |
| TLR3 | 5′-TGAGGCGGGTGTTTTTGAAC-3′ | 5′-GCATGATGTACCTTGAATCTTTTGC-3′ |
| HMGB3 | 5′-GACAGTCAGCCGCATCTTCT-3′ | 5′-GCGCCCAATACGACCAAATC-3′ |
| HMGB3-ChIPNC | 5'-CTCTAGGTACCCGCGGCCTG-3' | 5'-TGTGGGTGCCGCGGCCTTTG-3' |
| HMGB3-ChIP2  TGFβ-ChIP  TLR3-ChIP | 5'-CGTGGGACTGTCCTATGCAATG-3'  5'-GGAGGAGGATAACACAGAGA -3'  5'-CAAAGAAACAGTCTACCTCAG -3' | 5'-GAATGGGATTTGACTTGATCTC-3'  5'-ACAGCAGCCAAAGTGATC -3'  5'-GTCCCTCACATCTATCCATT -3' |

Table S2 The primer sequences applied in qPCR
