# Supplementary material for: TGIF2-mediated HMGB3 overexpression promotes esophageal squamous cell carcinoma proliferation and metastasis through TLR3/TGF-β signaling
Source: Genes Dis. 2025 Dec 15;13(3):101987. doi: 10.1016/j.gendis.2025.101987 (PMC12914543; doi:10.1016/j.gendis.2025.101987)
Supplement: Multimedia component 3 [file mmc3.docx]

| siNRA or shRNA | sequence |
| --- | --- |
| si-HMGB3-1 (stB0002760A) | CAGATAAAGTGCGCTATGA |
| si-HMGB3-2 (stB0002760B) | GTGCAAAGGGTCCTGCTAA |
| si-HMGB3-3 (stB0002760C) | GAGGCAAGAAGAAGAAGGA |
| si-TGF-β-1 (stB0003603A) | GCAAGACTATCGACATGGA |
| si-TGF-β-2 (stB0003603B) | GACACCAACTATTGCTTCA |
| si-TGF-β-3 (stB0003603C) | CACTGCAAGTGGACATCAA |
| shHMGB3-1 (93473-1) | CCGGCAGATAAAGTGCGCTATGACTCGAGTCATAGCGCACTTTATCTGTTTTTG |
| shHMGB3-2 (93474-1) | CCGGGAGGCAAGAAGAAGAAGGACTCGAGTCCTTCTTCTTCTTGCCTCTTTTTG |
| shTGIF2-1(94886-1) | CCGGCCATCCCTTTAGTCTCTGAAACTCGAGTTTCAGAGACTAAAGGGATGGTTTTT |
| shTGIF2-2(94887-1)  si-P65  si-TGIF2 | CCGGGACCCTAATCAGTTTACCATTCTCGAGAATGGTAAACTGATTAGGGTCTTTTT  CGGAUUGAGGAGAAACGUAAATT  GGCUGUACUUGCACCGCUA |

Table S3 The sequences of si-RNA or shRNA.
