# Supplementary material for: TGIF2-mediated HMGB3 overexpression promotes esophageal squamous cell carcinoma proliferation and metastasis through TLR3/TGF-β signaling
Source: Genes Dis. 2025 Dec 15;13(3):101987. doi: 10.1016/j.gendis.2025.101987 (PMC12914543; doi:10.1016/j.gendis.2025.101987)
Supplement: Multimedia component 4 [file mmc4.docx]

| GENE | HMGB3 | TLR3 |
| --- | --- | --- |
| Residue 1 | TYR-16 | VAL-720 |
| Residue 2 | LYS-10 | THR-710 |
| Residue 3 | LYS-174 | GLU-576 |

Table S4 The predicted binding interface residues between HMGB3 and TLR3
