# Supplementary material for: TGIF2-mediated HMGB3 overexpression promotes esophageal squamous cell carcinoma proliferation and metastasis through TLR3/TGF-β signaling
Source: Genes Dis. 2025 Dec 15;13(3):101987. doi: 10.1016/j.gendis.2025.101987 (PMC12914543; doi:10.1016/j.gendis.2025.101987)

Fig 1C

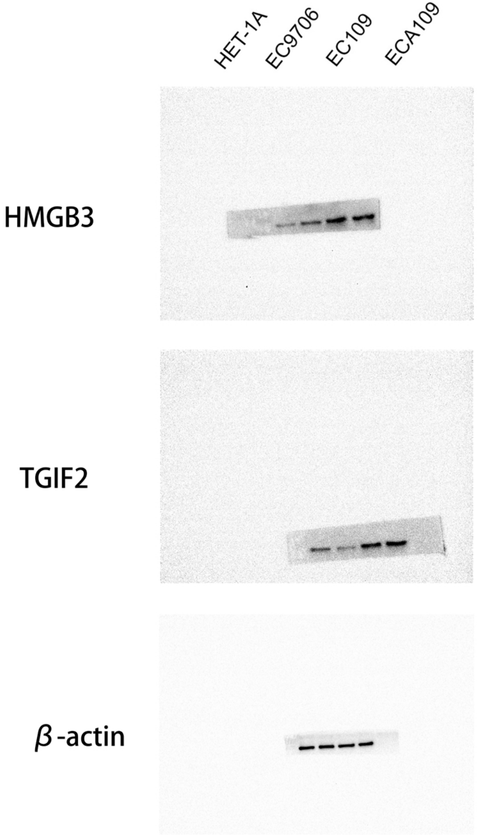

Fig4D

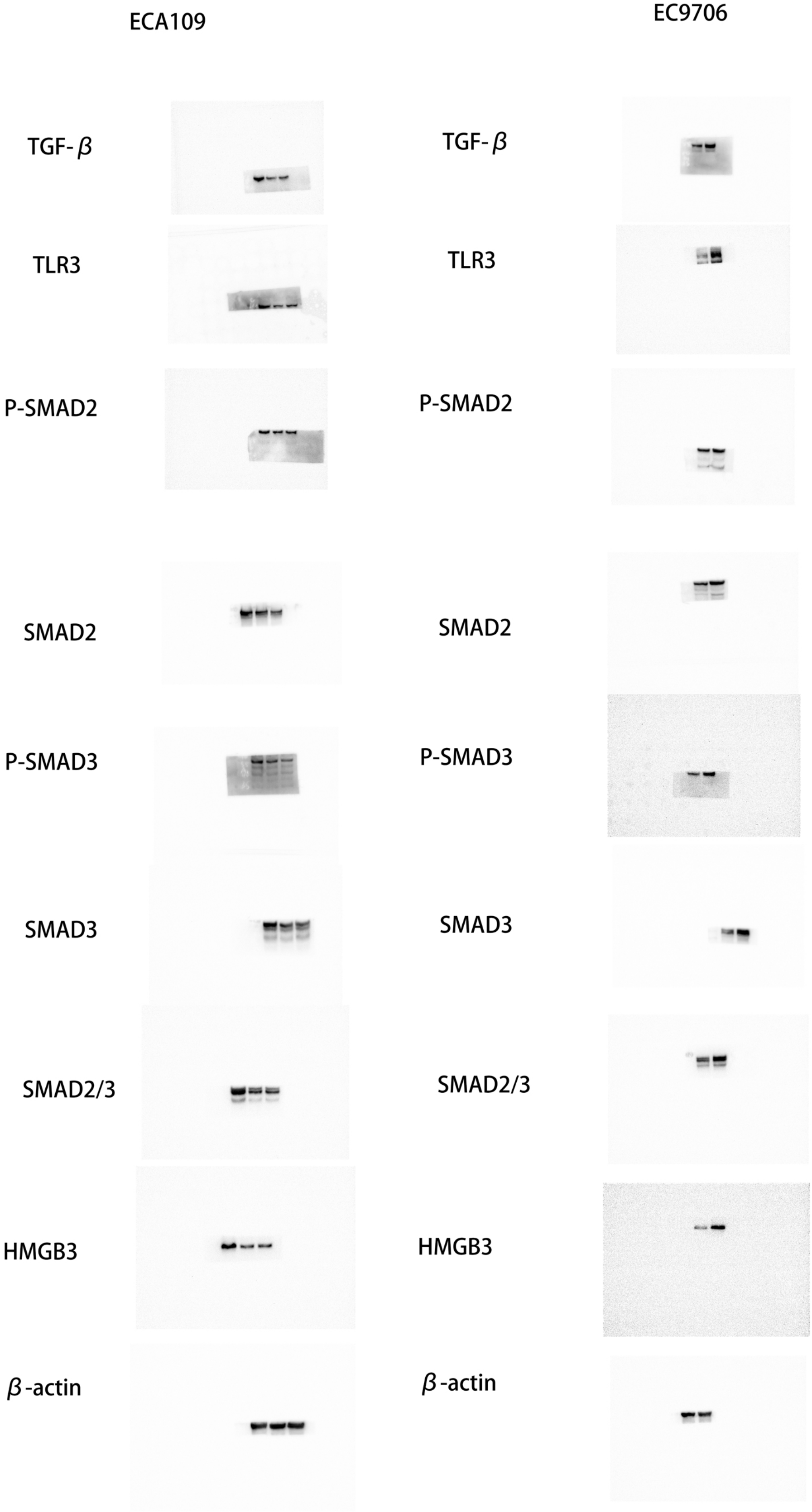
ECA109
EC9706
TGF-β
TGF-β
TLR3
TLR3
P-SMAD2
P-SMAD2
SMAD2
SMAD2
P-SMAD3
P-SMAD3
SMAD3
SMAD3
SMAD2/3
SMAD2/3
HMGB3
HMGB3
β-actin
β-actin

Fig 4G

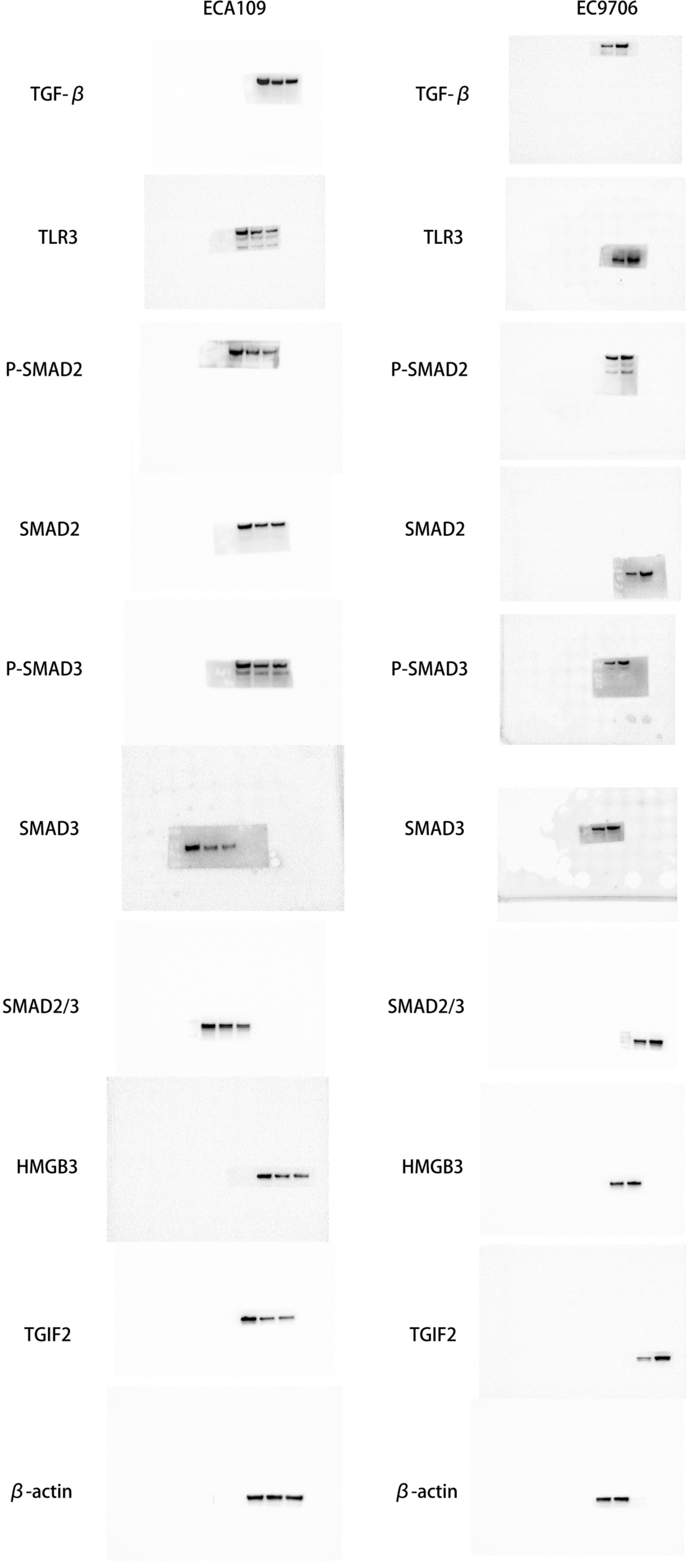
ECA109
EC9706
TGF-β
TGF-β
TLR3
TLR3
P-SMAD2
P-SMAD2
SMAD2
SMAD2
P-SMAD3
P-SMAD3
SMAD3
SMAD3
SMAD2/3
SMAD2/3
HMGB3
HMGB3
TGIF2
TGIF2
β-actin
β-actin

Fig4H

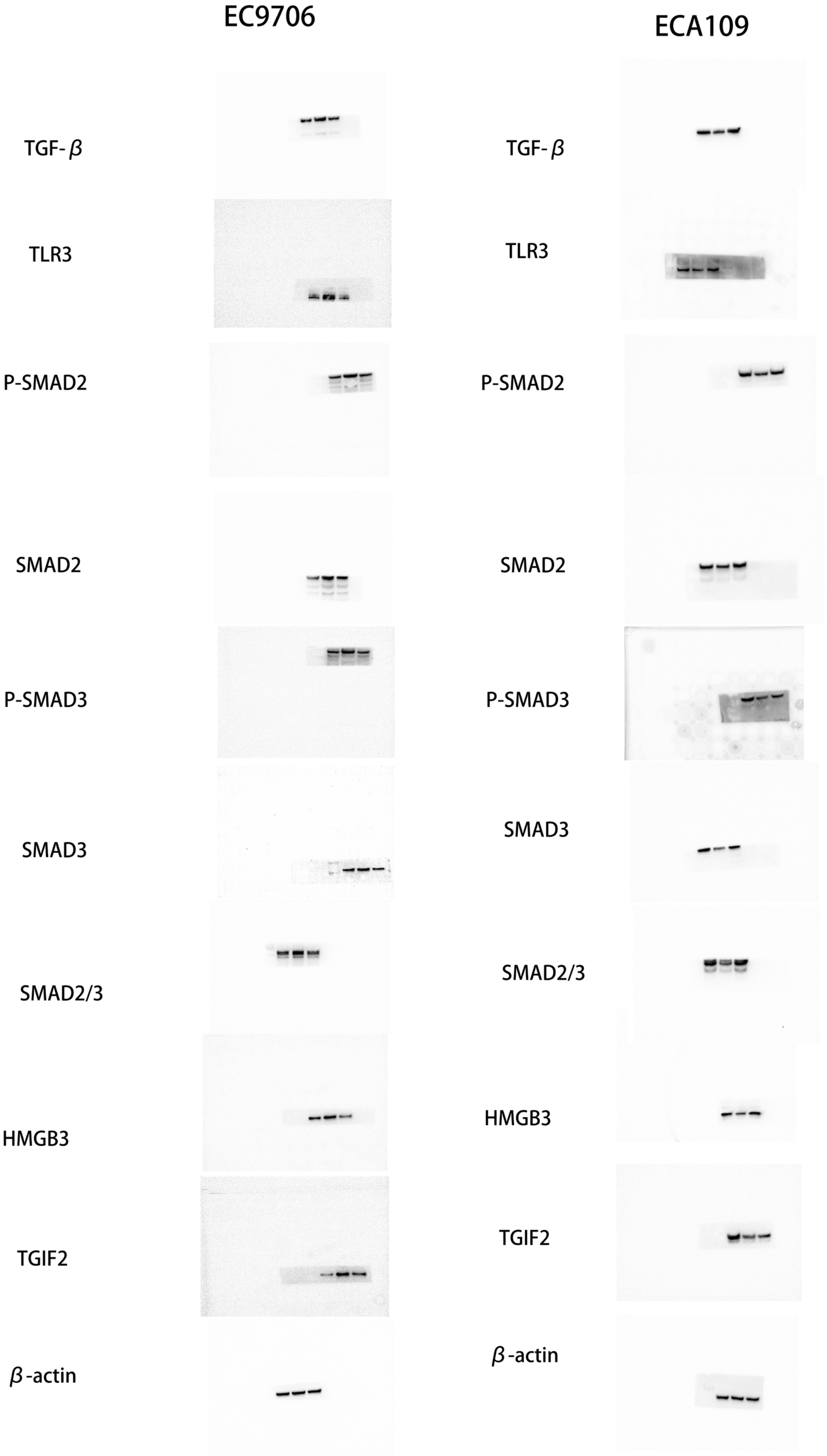

EC9706
ECA109
TGF-β
TGF-β
TLR3
TLR3
P-SMAD2
P-SMAD2
SMAD2
SMAD2
P-SMAD3
P-SMAD3
SMAD3
SMAD3
SMAD2/3
SMAD2/3
HMGB3
HMGB3
TGIF2
TGIF2
β-actin
β-actin

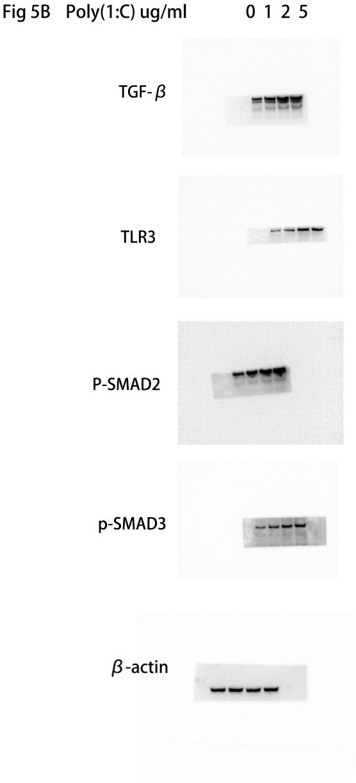

Fig 5B Poly(1:C) ug/ml
0 1 2 5
TGF-β
TLR3
P-SMAD2
p-SMAD3
β-actin

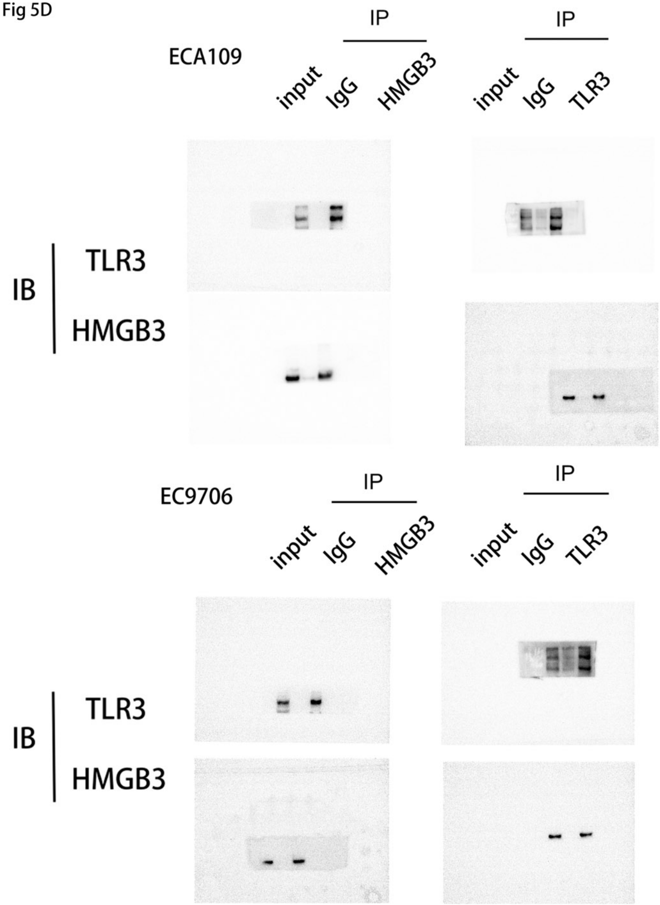

Fig 5D
ECA109
IP
IP
input
IgG
HMGB3
input
IgG
TLR3
IB
TLR3
HMGB3
EC9706
IP
IP
input
IgG
HMGB3
input
IgG
TLR3
IB
TLR3
HMGB3

Fig.6A

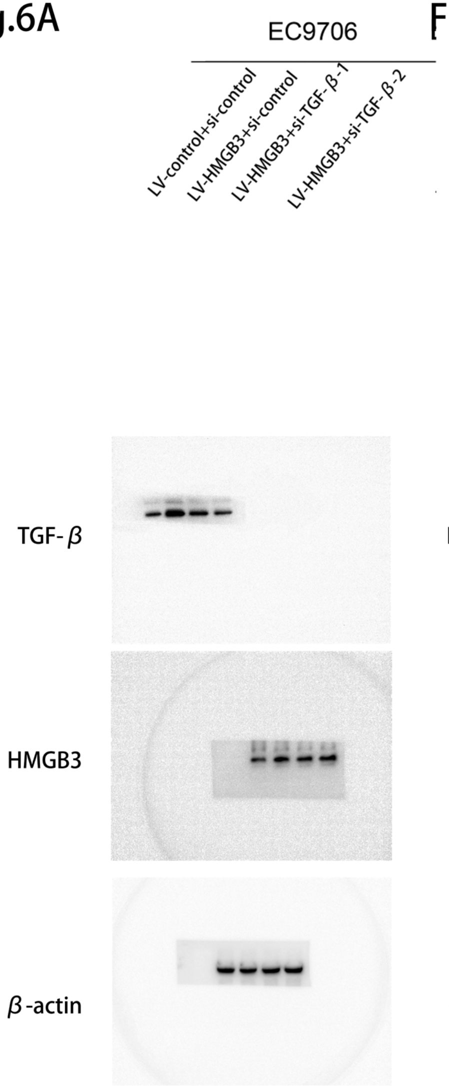

EC9706
LV-control+si-control
LV-HMGB3+si-control
LV-HMGB3+si-TGF-β-1
LV-HMGB3+si-TGF-β-2
TGF-β
HMGB3
β-actin

Fig.6B

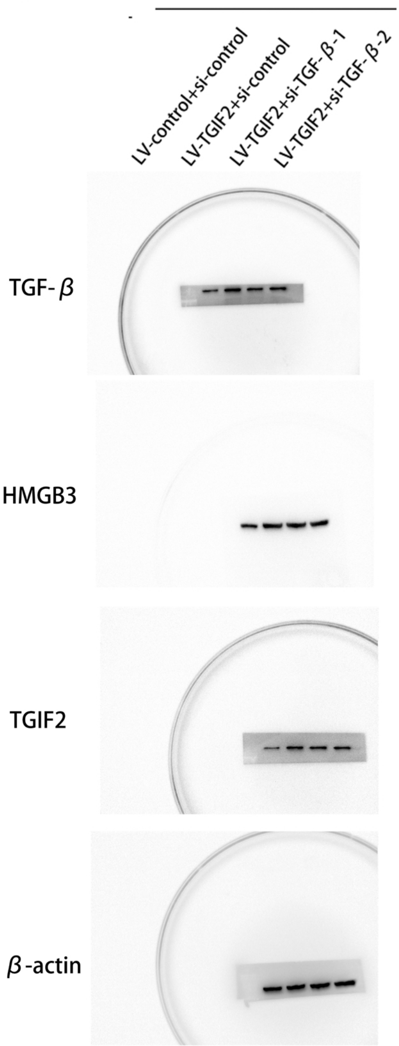

B
EC9706
LV-control+si-control
LV-TGIF2+si-control
LV-TGIF2+si-TGF-β-1
LV-TGIF2+si-TGF-β-2
TGF-β
HMGB3
TGIF2
β-actin

Fig.6G

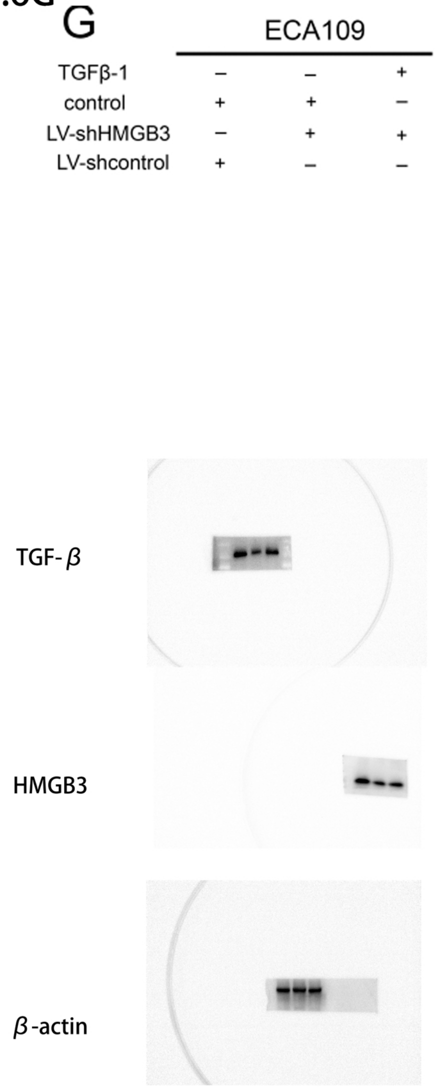
G
ECA109
TGFβ-1 – – +
control + + –
LV-shHMGB3 – + +
LV-shcontrol + – –
TGF-β
HMGB3
β-actin

Fig.6H

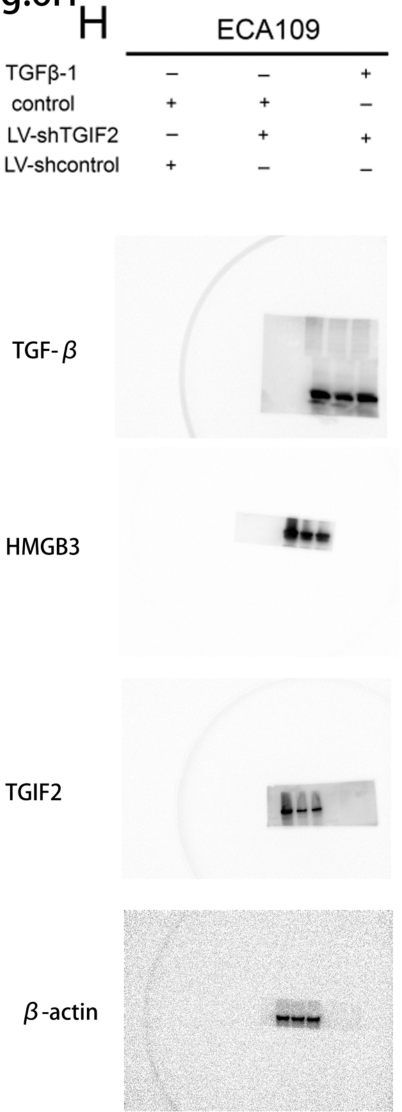
H
ECA109
TGFβ-1 – – +
control + + –
LV-shTGIF2 – + +
LV-shcontrol + – –
TGF-β
HMGB3
TGIF2
β-actin

Fig.6N

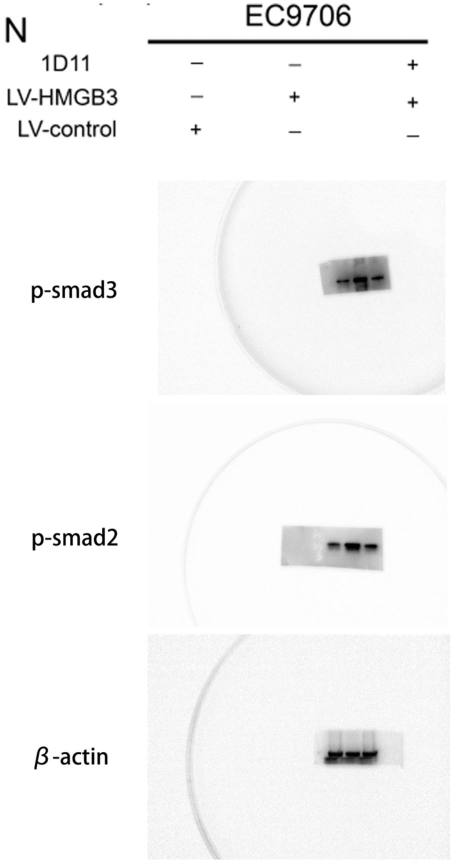

Supplement: Multimedia component 7 [file mmc7.pdf]
